# Abnormal epigenetic changes during differentiation of human skeletal muscle stem cells from obese subjects

**DOI:** 10.1186/s12916-017-0792-x

**Published:** 2017-02-22

**Authors:** Cajsa Davegårdh, Christa Broholm, Alexander Perfilyev, Tora Henriksen, Sonia García-Calzón, Lone Peijs, Ninna Schiøler Hansen, Petr Volkov, Rasmus Kjøbsted, Jørgen F. P. Wojtaszewski, Maria Pedersen, Bente Klarlund Pedersen, Dov B. Ballak, Charles A. Dinarello, Bas Heinhuis, Leo A. B. Joosten, Emma Nilsson, Allan Vaag, Camilla Scheele, Charlotte Ling

**Affiliations:** 1 Department of Clinical Sciences, Lund University Diabetes Centre, Lund University, Malmö, 205 02 Sweden; 2 Department of Endocrinology, Rigshospitalet, Copenhagen, 2100 Denmark; 3 The Centre of Inflammation and Metabolism and the Centre for Physical Activity Research, Rigshospitalet, University of Copenhagen, Copenhagen, Denmark; 4 Department of Exercise and Sports Sciences, Faculty of Health, University of Copenhagen, Copenhagen, Denmark; 5 Department of Integrative Physiology, University of Colorado Boulder, Boulder, CO 80309 USA; 6 Department of Medicine, University of Colorado, Aurora, CO 80045 USA; 7 Department of Internal Medicine, Radboud University Nijmegen Medical Centre, Nijmegen, The Netherlands; 8 Novo Nordisk Foundation Center for Basic Metabolic Research, University of Copenhagen, Copenhagen, Denmark; 9 Early Clinical Development, Translational Medical Unit, AstraZeneca, Mölndal, 431 83 Sweden

**Keywords:** DNA methylation, Myogenesis, Obesity, IL-32, Epigenetics, ARPP21, TGF-β3, PSG, CGB, MT, Insulin resistance

## Abstract

**Background:**

Human skeletal muscle stem cells are important for muscle regeneration. However, the combined genome-wide DNA methylation and expression changes taking place during adult myogenesis have not been described in detail and novel myogenic factors may be discovered. Additionally, obesity is associated with low relative muscle mass and diminished metabolism. Epigenetic alterations taking place during myogenesis might contribute to these defects.

**Methods:**

We used Infinium HumanMethylation450 BeadChip Kit (Illumina) and HumanHT-12 Expression BeadChip (Illumina) to analyze genome-wide DNA methylation and transcription before versus after differentiation of primary human myoblasts from 14 non-obese and 14 obese individuals. Functional follow-up experiments were performed using siRNA mediated gene silencing in primary human myoblasts and a transgenic mouse model.

**Results:**

We observed genome-wide changes in DNA methylation and expression patterns during differentiation of primary human muscle stem cells (myoblasts). We identified epigenetic and transcriptional changes of myogenic transcription factors (*MYOD1*, *MYOG*, *MYF5*, *MYF6*, *PAX7*, *MEF2A*, *MEF2C*, and *MEF2D*), cell cycle regulators, metabolic enzymes and genes previously not linked to myogenesis, including *IL32*, metallothioneins, and pregnancy-specific beta-1-glycoproteins. Functional studies demonstrated IL-32 as a novel target that regulates human myogenesis, insulin sensitivity and ATP levels in muscle cells. Furthermore, *IL32* transgenic mice had reduced insulin response and muscle weight. Remarkably, approximately 3.7 times more methylation changes (147,161 versus 39,572) were observed during differentiation of myoblasts from obese versus non-obese subjects. In accordance, *DNMT1* expression increased during myogenesis only in obese subjects. Interestingly, numerous genes implicated in metabolic diseases and epigenetic regulation showed differential methylation and expression during differentiation only in obese subjects.

**Conclusions:**

Our study identifies IL-32 as a novel myogenic regulator, provides a comprehensive map of the dynamic epigenome during differentiation of human muscle stem cells and reveals abnormal epigenetic changes in obesity.

**Electronic supplementary material:**

The online version of this article (doi:10.1186/s12916-017-0792-x) contains supplementary material, which is available to authorized users.

## Background

Human skeletal muscle stem cells, called satellite cells, are quiescent, but activated in response to muscle injury [1] and resistance exercise training [2]. Activation of satellite cells represents a muscle repair mechanism. Upon activation, these cells proliferate and a subpopulation, myoblasts, differentiates and fuses with existing muscle fibers or other differentiating muscle cells [3], thus contributing to multinucleated muscle fibers. The process, called myogenesis, is strictly controlled by timely expression of a network of myogenic regulatory factors (MRFs), including MYOD, MYF5, MYOG, and MYF6, together with myocyte enhancer factors 2 (MEF2s) [4, 5]. However, it is possible that novel myogenic factors that regulate differentiation of human myoblasts into myotubes, and consequently muscle cell function, can be discovered. Additionally, although epigenetic mechanisms such as DNA methylation are known to regulate cell differentiation and cell-specific gene expression, the epigenome and transcriptome of human adult myogenesis is far from defined [6–8].

Obesity is a major risk factor for developing muscular insulin resistance, a condition preceding type 2 diabetes [9], and is associated with decreased relative muscle mass and impaired muscle metabolism [10, 11]. Satellite cells reside under the basal lamina of the muscle fibers and are thus exposed to the same environmental factors as the muscle fibers. We have previously reported that in vivo properties, including decreased insulin sensitivity and growth factor signaling, are preserved when human satellite cells from obese subjects with type 2 diabetes are isolated and differentiated to myotubes in vitro [12–14]. Therefore, we hypothesized that obesity-dependent epigenetic modifications are established in human satellite cells and potentially affect myogenesis in obese individuals.

Of note, factors such as high fat diet, exercise, and aging have previously been shown to alter the DNA methylation pattern in human skeletal muscle [15–19]. Additionally, muscle biopsies isolated from monozygotic twin pairs discordant for type 2 diabetes and body mass index (BMI) exhibit differential DNA methylation [20].

Based on this background, we aimed to study changes in the genome-wide DNA methylation and gene expression patterns during human adult myogenesis, i.e. differentiation of activated primary human muscle stem cells (myoblasts) from healthy subjects into myotubes, and also examine if obesity affects the epigenetic and transcriptional changes that take place during this process. To address these questions we (1) studied changes in the genome-wide DNA methylation and expression patterns during differentiation of primary human myoblasts into myotubes in healthy subjects; (2) performed functional follow-up experiments of some identified candidate genes previously not known to affect myogenesis in order to find novel targets that regulate muscle regeneration and function; and (3) examined if the epigenetic and transcriptional changes that take place in human myoblasts during differentiation into myotubes are different in obese compared with non-obese subjects.

## Methods

### Primary human muscle stem cell isolation and culture of myoblasts

Primary muscle stem cells (satellite cells) were isolated from human vastus lateralis biopsies. Myoblasts were harvested when less than 50% confluent (Additional file 1: Figure S1A). See Additional file 1: Supplemental Methods for details of myoblast culture and differentiation.

### DNA methylation and mRNA expression arrays

DNA methylation was analyzed using Infinium HumanMethylation450 BeadChip (Illumina, San Diego, CA, USA). mRNA expression was analyzed using HumanHT-12 Expression BeadChip (Illumina). See Additional file 1: Supplemental Methods for details.

### Statistical analysis

Two-tailed t-tests were used to compare clinical characteristics between the groups. Comparison of DNA methylation and expression data between myoblasts and myotubes were analyzed with paired Wilcoxon signed rank tests and for array data adjusted for multiple testing with false discovery rate (FDR) analysis. Spearman correlations were used to correlate gene expression and DNA methylation. Number of correlations expected by chance was calculated as number of tests times 0.05. Data from gene silencing experiments were analyzed with paired t-tests and log-transformed values were used for qPCR data. Mouse data were analyzed with Mann–Whitney U tests. Distributions were analyzed with χ^2^ tests and Bonferroni-corrected when feasible. To compare DNA methylation and mRNA expression array data between non-obese and obese subjects we used a linear regression analysis including obesity, age, and sex as co-variates. A FDR less than 5% (*q* < 0.05) was applied. See Additional file 1: Supplemental Methods for more details of materials and methods.

## Results

### Human muscle stem cells

Human muscle stem cells (satellite cells) were isolated from skeletal muscle biopsies obtained from 14 healthy non-obese and 14 obese subjects. Their clinical characteristics are described in Table 1. Isolated satellite cells were expanded and differentiated from myoblasts into myotubes [13]. Cells were harvested as proliferating myoblasts and as fully differentiated myotubes (Additional file 1: Figure S1A).

**Table 1:** Clinical characteristics of study participants

	Non-obese (*N* = 14)	Obese (*N* = 14)	*P* value
Men/Women	7/7	8/6	
Age, years	54.2 ± 6.8	50.2 ± 6.2	1.3 × 10^−1^
BMI, kg/m^2^	24.7 ± 2.4	35.1 ± 2.8	5.1 × 10^−11^
Weight, kg	74.5 ± 14.2	111.3 ± 9.0	1.2 × 10^−9^
Hip circumference, cm	100.6 ± 4.7	119.6 ± 6.9	5.7 × 10^−9^
Waist circumference, cm	85.9 ± 10.2	116.1 ± 8.6	6.2 × 10^−9^
Android fat mass, kg	1.7 ± 7.1	4.9 ± 1.0	4.3 × 10^−10^
Gynoid fat mass, kg	4.0 ± 1.3	7.0 ± 2.0	6.5 × 10^−5^
Whole body fat mass, kg	20.0 ± 6.0	42.7 ± 8.7	1.7 × 10^−8^
Whole body fat-free mass, kg	51.4 ± 13.3	63.9 ± 11.9	1.4 × 10^−2^
Fasting glucose, mmol/L	4.8 ± 0.6	5.0 ± 0.4	2.3 × 10^−1^
2-h glucose, mmol/L^a^	5.2 ± 1.4	5.5 ± 1.4	5.6 × 10^−1^
Fasting insulin, pmol/L	32.1 ± 13.3	92.7 ± 30.3	3.8 × 10^−7^
2-h insulin, pmol/L^a^	230.1 ± 155.3	441.9 ± 287.0	2.4 × 10^−2^
HOMA-IR	1.0 ± 0.5	3.0 ± 1.1	1.8 × 10^−6^
HOMA-β, %	74.4 ± 26.7	184.4 ± 74.8	2.4 × 10^−5^
P-Cholesterol-total, mmol/L	5.5 ± 1.0	5.2 ± 0.8	4.3 × 10^−1^
P-Cholesterol-HDL, mmol/L	1.7 ± 0.5	1.3 ± 0.3	1.7 × 10^−2^
P-Cholesterol-LDL, mmol/L	3.3 ± 0.9	3.3 ± 0.6	9.8 × 10^−1^
Systolic blood pressure, mmHg	134.3 ± 12.0	138.5 ± 18.1	4.7 × 10^−1^
Diastolic blood pressure, mmHg	85.6 ± 9.5	91.6 ± 9.1	9.8 × 10^−1^
Pulse, beats/min	59.5 ± 12.4	66.6 ± 8.4	8.6 × 10^−11^
VO_2_ max per kg, mL/min/kg	32.6 ± 11.4	22.2 ± 4.4	3.8 × 10^−3^

Data are presented as mean ± SD. Two-tailed t-tests were used for statistical calculations

^a^Measured during a 2-h oral glucose tolerance test

To evaluate myogenic purity of the cultured cells, we assessed their cell surface marker expression by flow cytometry analysis. To discriminate myogenic cells from non-myogenic cells, we used CD56, which is expressed on myogenic cells, and CD31 and CD45, which are expressed on endothelial and hematopoietic cells, respectively [21]. All cell cultures were negative for CD31 and CD45 and all cell cultures were positive for CD56; there was no significant difference between the non-obese and obese groups in the percentage of gated single cells expressing CD56 (Additional file 1: Figure S1B–D).

### Changes in the global DNA methylation pattern during differentiation of primary human myoblasts into myotubes of healthy subjects

**Fig. 1:**
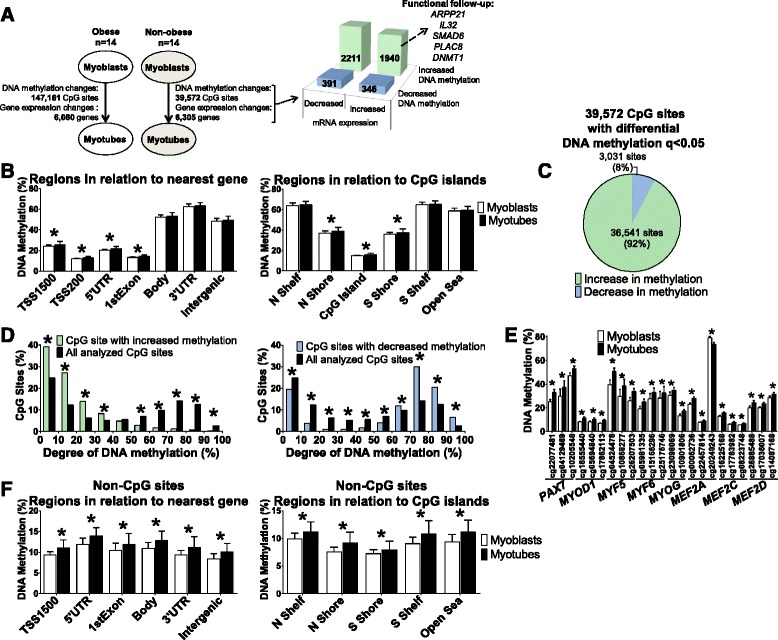
The human methylome before versus after differentiation of human primary myoblasts. **a** An illustrative description of the study design and comparisons performed in cells of both non-obese and obese subjects. **b** Global DNA methylation was calculated as the average degree of methylation for all analyzed CpG sites before (*n* = 14) and after (*n* = 14) differentiation of myoblasts of non-obese subjects based on Illumina’s annotations to different gene regions and CpG island regions. Data are presented as mean ± SEM. Total number of sites per region: TSS1500, 83,650; TSS200, 62,369; 5’UTR, 64,909; 1^st^ Exon, 39,236; Body, 172,275; 3’UTR, 19,349; Intergenic, 116,209. **c** A pie chart showing the number and proportions of individual CpG sites with significantly increased and decreased methylation, respectively, in human myoblasts compared with myotubes (*q* < 0.05) of non-obese subjects (*n* = 14). **d** Proportions of significant CpG sites with a certain degree of methylation before differentiation compared with the proportions of all analyzed sites. **e** DNA methylation of the three most significant CpG sites annotated to key myogenic transcription factors in human myoblasts versus myotubes of non-obese subjects. **f** Global DNA methylation was calculated as the average degree of methylation for all analyzed non-CpG sites before and after differentiation in non-obese subjects based on Illumina’s annotations to different gene region and CpG island regions. Data are presented as mean ± SEM. TSS, proximal promoter, defined as 200 bp (base pairs) or 1500 bp upstream of transcription start site; UTR, untranslated region; CpG island, 200 bp (or more) stretch of DNA with a C + G content greater than 50% and an observed CpG/expected CpG in excess of 0.6; Shore, the flanking region of CpG islands, 0–2000 bp; Shelf, regions flanking island shores, i.e., covering 2000–4000 bp distant from the CpG island. * *q* < 0.05

To examine if epigenetic changes take place during human myogenesis, we examined the genome-wide DNA methylation pattern in human myoblasts and myotubes from 14 healthy non-obese subjects using the Infinium HumanMethylation450 BeadChip. An overview of comparisons performed in this study is presented in Fig. 1a.

After quality control, DNA methylation data were obtained for a total of 477,226 CpG sites in myoblasts and myotubes. The analyzed CpG sites have been annotated to different gene regions based on their genomic location and in relation to CpG islands [22]. To examine the global methylation level for myoblasts and myotubes, we calculated the average level of DNA methylation in these gene regions (Fig. 1b). The highest level of methylation (48–63%) was found distant from promoter regions (gene body and 3’UTR) and in intergenic regions, whereas regions close to the transcription start site (TSS1500, TSS200, 5‘UTR and 1^st^ exon) were least methylated (12–26%). Interestingly, the average methylation levels for TSS1500, TSS200, 5’UTR and 1^st^ exon were significantly increased after differentiation (q < 0.05). When investigating global methylation in CpG island regions, the CpG islands (15%) and the shores (36–37%) were least methylated and their average methylation levels increased significantly after differentiation (q < 0.05). The shelves and the open sea had an average methylation level of approximately 60% and were not altered during myogenesis. Of note, all genomic regions with a relatively low degree of methylation increased significantly after differentiation, suggesting that epigenetic regulation in these regions might play an important role during human myogenesis.

### Changes in DNA methylation of individual CpG sites during differentiation of primary human myoblasts into myotubes of healthy subjects

We next tested if DNA methylation of any individual CpG site changed in myoblasts versus myotubes from the 14 healthy non-obese subjects. Indeed, DNA methylation of a large number of individual CpG sites (39,572) changed significantly during differentiation (FDR less than 5%; q < 0.05). The significant sites are annotated to 13,639 unique genes and intergenic regions (Additional file 2: Table S1). Our findings reflect dominant de novo methylation as the majority of these sites (92%, 36,541 sites) had increased methylation, whereas only 8% (3031 sites) had decreased methylation after differentiation (Fig. 1c). CpG sites with increased methylation after differentiation generally had a low degree of methylation in myoblasts, whereas CpG sites with decreased methylation had a relatively high degree of methylation in myoblasts (Fig. 1d). The largest absolute change in methylation and fold-change were 16.5% and 2.05, respectively (Additional file 2: Table S1).

Interestingly, several key myogenic transcription factors, including *PAX7*, MRFs, and MEF2s, had numerous CpG sites with significant changes in DNA methylation before versus after differentiation (Additional file 2: Table S1), suggesting that changes in DNA methylation contribute to the regulation of human myogenesis. The three most significant CpG sites for each of these myogenic transcription factors are shown in Fig. 1e. Of note, *PAX7* showed differential methylation of 24 of 81 analyzed CpG sites, all but one with increased methylation, and 7 of 9 analyzed CpG sites annotated to *MYF5* showed significantly increased methylation after differentiation.

We next performed KEGG pathway analysis using Webgestalt [23] to identify biological pathways with enrichment of genes that exhibited differential DNA methylation during myogenesis. Genes with one or more differentially methylated CpG site(s) were significantly enriched in 27 KEGG-pathways (Table 2 and Additional file 2: Table S1). Among the significant pathways were MAPK signaling, cell cycle, oxidative phosphorylation, regulation of actin cytoskeleton, TGF-β signaling, and calcium signaling, together supporting the development of a functional myotube [4].

**Table 2:** Significantly enriched KEGG pathways among differentially methylated genes between myoblasts and myotubes

Pathway (total number of genes in pathway)	Observed/Expected number of genes	*P* value	*q* value
Non-obese subjects			
Pathways in cancer (325)	321/295	9.8 × 10^−10^	2.2 × 10^−7^
Metabolic pathways (1112)	1060/1009	3.3 × 10^−9^	3.7 × 10^−7^
Focal adhesion (200)	199/182	7.6 × 10^−8^	4.3 × 10^−6^
Endocytosis (200)	199/182	7.6 × 10^−8^	4.3 × 10^−6^
MAPK signaling pathway (267)	260/242	1.4 × 10^−5^	5.0 × 10^−4^
Ubiquitin mediated proteolysis (134)	133/122	3.3 × 10^−5^	1.1 × 10^−3^
Neurotrophin signaling pathway (127)	126/115	6.2 × 10^−5^	1.8 × 10^−3^
Protein processing in endoplasmic reticulum (165)	162/150	1.0 × 10^−4^	1.8 × 10^−3^
Lysosome (121)	120/110	1.0 × 10^−4^	1.8 × 10^−3^
Cell cycle (124)	123/113	8.1 × 10^−5^	1.8 × 10^−3^
Oxidative phosphorylation (119)	118/108	1.0 × 10^−4^	1.8 × 10^−3^
Regulation of actin cytoskeleton (211)	205/192	2.0 × 10^−4^	2.9 × 10^−3^
TGF-beta signaling pathway (84)	84/76	3.0 × 10^−4^	3.8 × 10^−3^
Axon guidance (129)	127/117	4.0 × 10^−4^	4.8 × 10^−3^
Phosphatidylinositol signaling system (78)	78/71	5.0 × 10^−4^	5.7 × 10^−3^
Purine metabolism (162)	158/147	6.0 × 10^−4^	6.5 × 10^−3^
Calcium signaling pathway (176)	171/160	8.0 × 10^−4^	7.9 × 10^−3^
Pyrimidine metabolism (98)	97/89	8.0 × 10^−4^	7.9 × 10^−3^
Adipocytokine signaling pathway (68)	68/62	1.4 × 10^−3^	1.3 × 10^−2^
p53 signaling pathway (68)	68/62	1.4 × 10^−3^	1.3 × 10^−2^
Wnt signaling pathway (149)	145/135	1.5 × 10^−3^	1.3 × 10^−2^
ErbB signaling pathway (87)	86/79	2.1 × 10^−3^	1.7 × 10^−2^
Melanogenesis (101)	99/92	3.5 × 10^−3^	2.6 × 10^−2^
Inositol phosphate metabolism (57)	57/52	4.0 × 10^−3^	2.9 × 10^−2^
Peroxisome (79)	78/72	4.3 × 10^−3^	3.0 × 10^−2^
mTOR signaling pathway (52)	52/47	6.5 × 10^−3^	4.2 × 10^−2^
Spliceosome (127)	123/115	7.0 × 10^−3^	4.4 × 10^−2^
Obese subjects			
Pathways in cancer (325)	325/311	4.3 × 10^−7^	9.8 × 10^−5^
Metabolic pathways (1112)	1086/1063	2.0 × 10^−4^	2.3 × 10^−2^

MicroRNA (miRNA) is another epigenetic mechanism known to regulate gene transcription and DNA methylation may further regulate miRNA expression [24]. A number of miRNAs has also been shown to influence myogenesis [25]. Here, we found significant methylation changes of 225 CpG sites annotated to 157 different miRNA genes during myogenesis (Additional file 2: Table S1), including 17 of the 60 miRNA identified by Dmitriev et al. [25].

### Methylation of non-CpG sites during myogenesis

Although DNA methylation in the human genome mainly takes place on cytosines in CpG sites, cytosines followed by another base than guanine can also be methylated, by the so called non-CpG methylation. Methylation of non-CpG sites has previously been found in stem cells [26] and we therefore tested if methylation of these sites changed during myogenesis. The average methylation level of the analyzed non-CpG sites increased significantly for all gene and CpG island regions after differentiation (Fig. 1f). Additionally, methylation of 665 individual non-CpG sites (60% of the analyzed sites) changed significantly and all showed increased methylation after differentiation (*q* < 0.05) (Additional file 2: Table S1).

### Changes in the transcriptome during differentiation of primary human myoblasts into myotubes of healthy subjects

We proceeded to evaluate the genome-wide transcriptome changes characterizing human myogenesis. Complete mRNA data were obtained from 13 healthy non-obese subjects. We found significant expression changes of a large number of transcripts (7699 with FDR < 5%) corresponding to 6305 unique genes before versus after differentiation (Additional file 3: Table S2); 45% of these transcripts were upregulated and 55% were downregulated.

**Fig. 2:**
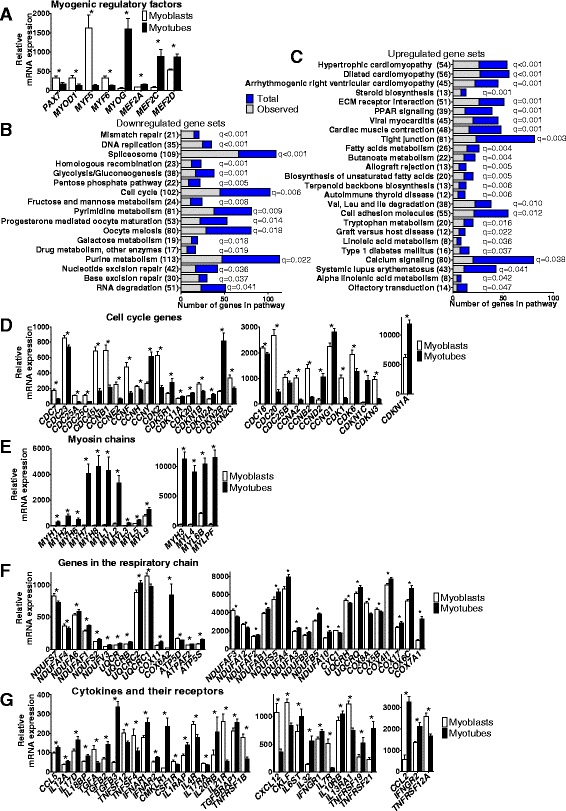
Transcriptional changes before versus after differentiation of human primary myoblasts of non-obese subjects. **a** Significant changes in mRNA expression of key myogenic transcription factors in human myoblasts (*n* = 13) versus myotubes (*n* = 13) of non-obese subjects. Significantly enriched downregulated (**b**) and upregulated (**c**) gene sets in Gene Set Enrichment Analysis with FDR < 5% (*q* < 0.05) based on differential gene expression in human myoblasts versus myotubes of non-obese subjects. Significant changes in mRNA expression of genes encoding cell cycle proteins (**d**), myosin heavy and light chains (**e**), proteins in the respiratory chain (**f**), and cytokines and their receptors (**g**) in human myoblasts versus myotubes of non-obese subjects (*n* = 13). Data are presented as mean ± SEM. ECM, Extracellular matrix; Val, Valine; Leu, Leucine; Ile, Isoleucine. * *q* < 0.05

Importantly, several key myogenic transcription factors were differentially expressed between myoblasts and myotubes (Fig. 2a). For example, *PAX7*, *MYOD1*, *MYF5*, and *MYF6* showed significantly decreased expression, while *MYOG*, *MEF2A*, *MEF2C*, and *MEF2D* showed increased expression after differentiation (Fig. 2a and Additional file 3: Table S2). In accordance, all of these genes exhibited differential DNA methylation during myogenesis (Fig. 1e and Additional file 2: Table S1).

To further dissect what cellular events the differentially expressed genes are involved in, Gene Set Enrichment Analysis (GSEA) [27] was applied. Among downregulated genes, we identified 17 significant gene sets, including gene sets related to DNA replication and nucleotide biosynthesis, i.e., the cell cycle (Fig. 2b and Additional file 3: Table S2). Additionally, 25 gene sets were significant among upregulated genes, including extracellular matrix receptor interactions, calcium signaling, and several pathways involved in cellular metabolism (Fig. 2c and Additional file 3: Table S2), altogether confirming that the cells developed from proliferating myoblasts into metabolically active myotubes (Additional file 1: Figure S1A). Notably, many genes regulating the cell cycle (Fig. 2d) and protein turnover showed significantly altered expression during myogenesis, reflecting withdrawal from the cell cycle before differentiation. The negative regulators of the cell cycle, *CDKN1A* and *CDKN1C*, were upregulated while many cyclins (*CCNA2*, *CCNB1*, *CCNB2*, and *CCNE2*) and cyclin-dependent kinases (*CDK1*, *CDK2*, and *CDK6)* were downregulated after differentiation. Moreover, numerous genes important for muscle contraction, such as myosin light chain (MLC) and heavy chain (MHC) (Fig. 2e), troponin, and actin, genes regulating storage, release and reuptake of calcium ions, as well as genes involved in oxidative phosphorylation (Fig. 2f) were upregulated after differentiation.

Since skeletal muscle is an endocrine organ [28] we also examined if the expression pattern of a number of cytokines, growth factors, and their receptors changed during differentiation (Fig. 2g). The regulation pattern of these genes may reflect autocrine properties of skeletal muscle cells, which are crucial for muscle repair and development [1].

### Identification of gene families with novel regulation patterns during myogenesis

**Fig. 3:**
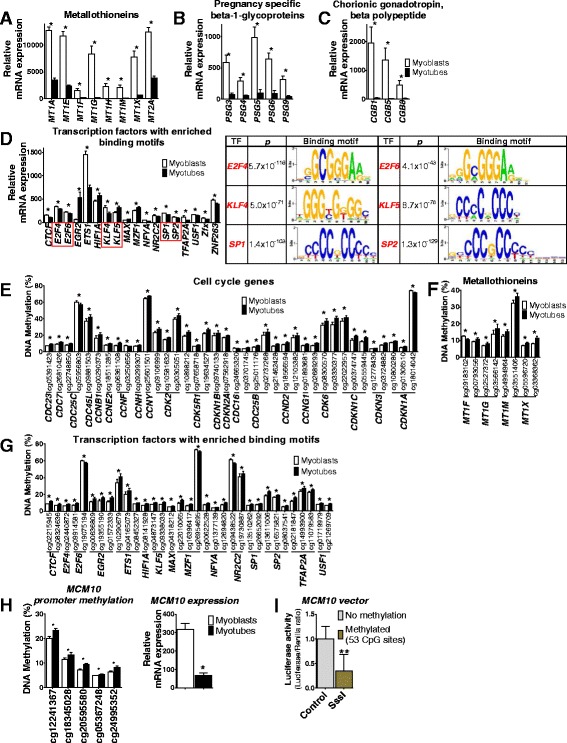
Newly identified gene families, transcription factor binding motifs, and overlapping changes in gene expression and DNA methylation before versus after differentiation of human primary myoblasts of non-obese subjects. Significant changes in mRNA expression of genes encoding metallothioneins (**a**), pregnancy-specific beta-1 glycoproteins (**b**), and chorionic gonadotropin beta polypeptides (**c**) in human myoblasts versus myotubes of non-obese subjects (*n* = 13). **d** To the left is mRNA expression of identified transcription factors with significant enrichment of binding motifs in promoter regions of all differentially expressed genes in human myoblasts versus myotubes of non-obese subjects (*n* = 13). Those with a red box are further emphasized to the right in the figure. This figure shows Bonferroni adjusted *P* values and binding motifs for enriched transcription factors in the SP/KLF family and E2F transcription factor family. DNA methylation of the three most significant CpG sites in myoblasts versus myotubes annotated to genes that also showed differential mRNA expression among cell cycle genes (**e**), metallothioneins (**f**), and transcription factors with enriched binding motifs among all differentially expressed genes (**g**) in non-obese subjects (*n* = 14). (See also Figs. 2d, 3a and d). **h** Increased promoter methylation and reduced expression of *MCM10* during differentiation. **i** In vitro methylation of the *MCM10* promoter resulted in decreased transcriptional activity, measured as luciferase activity (*n* = 4, **P < 0.01). Data are presented as mean ± SEM. TF, Transcription factor. * *q* < 0.05

Remarkably, we also identified several gene families previously not known to affect myogenesis, including metallothioneins, pregnancy-specific beta-1-glycoproteins (PSGs), and chorionic gonadotropin beta polypeptides (CGBs) (Fig. 3a–c). Interestingly, all significant genes in these families were highly expressed in myoblasts and strongly downregulated after differentiation. While two of the gene families, PSG and CGB, are produced by trophoblasts during pregnancy [29, 30], their expression has never been reported in myoblasts.

### Identification of transcription factor binding motifs of importance for myogenesis

To identify transcription factors potentially driving transcriptional changes during myogenesis, we used PSCAN [31] and JASPAR [32]. Here, we searched for overrepresentation of specific transcription factor binding sequences among the differentially expressed genes. Recognition sequences for 48 transcription factors were significantly enriched (*P*_Bonferroni_ < 0.05) (Additional file 3: Table S2) and 18 of these did also themselves show differential expression between myoblasts and myotubes (Fig. 3d), including members in the SP/KLF family and E2F transcription factor family.

### Overlapping changes in DNA methylation and mRNA expression during myogenesis

Epigenetic modifications can affect gene expression [33]; hence, we looked for genes that had changes in both DNA methylation and expression after differentiation. When comparing myoblasts and myotubes from healthy subjects, 4285 genes had significant differences in both expression and DNA methylation of one or more CpG site(s) (Fig. 1a, Table 3 and Additional file 4: Table S3). These include the MRFs (Figs. 1e and 2a), many of the cell cycle genes (Figs. 2d and 3e), metallothioneins (Fig. 3a and f), and the transcription factors identified with PSCAN (Fig. 3d and g).

**Table 3:** Twenty genes with largest fold-change in expression that were also differentially methylated at one or more CpG site before versus after differentiation in non-obese

Expression						DNA methylation (%)					
IlluminaID	Gene symbol	Myoblasts	Myotubes	Ratio	*q* value	Probe ID	Gene region	Myoblasts	Myotubes	Difference	*q* value
Downregulated											
1170300	*MT1G*	8314.1 ± 5678.0	167.0 ± 63.3	0.02	0.0017	cg03566142	5′UTR;1stExon	13.8 ± 2.5	17.2 ± 2.6	3.4	0.033
3710161	*LHB*	547.1 ± 606.8	18.3 ± 9.7	0.03	0.0017	cg06452915	TSS1500	8.8 ± 0.4	12.2 ± 1.1	3.5	0.029
1400634	*MT1M*	2099.9 ± 1709.1	70.5 ± 33.9	0.03	0.0017	cg04994964	5′UTR;1stExon	12.7 ± 0.7	15.2 ± 1.1	2.5	0.026
4880609	*DKK1*	767.1 ± 567.5	73.6 ± 57.5	0.1	0.0025	cg11988964	TSS1500	13.4 ± 1.1	19.3 ± 1.7	5.9	0.026
						cg02302582	TSS1500	15.8 ± 1.6	20.0 ± 1.5	4.2	0.031
						cg27411220	TSS1500	11.0 ± 2.1	14.5 ± 2.8	3.5	0.033
						cg02493604	TSS1500	12.6 ± 1.6	17.0 ± 2.3	4.4	0.041
						cg07684796	1stExon	7.6 ± 0.4	10.3 ± 1.4	2.6	0.049
6660601	*HMOX1*	3277.5 ± 1728.4	209.7 ± 104.1	0.1	0.0017	cg05718255	3′UTR	17.9 ± 2.3	22.4 ± 1.9	4.5	0.049
6620528	*MT1X*	7739.1 ± 4215.6	624.4 ± 155.5	0.1	0.0017	cg03551406	TSS1500	32.2 ± 2.0	36.1 ± 2.3	3.9	0.026
						cg03368362	TSS1500	8.0 ± 0.4	11.5 ± 1.1	3.5	0.033
						cg05596720	TSS200	6.8 ± 0.3	8.6 ± 0.4	1.7	0.026
2480452	*MYF5*	1631.4 ± 1200.0	130.9 ± 77.6	0.1	0.0017	cg14952949	TSS1500	25.1 ± 3.2	32.8 ± 2.6	7.7	0.029
						cg04524478	TSS1500	39.6 ± 4.1	50.7 ± 3.0	11.1	0.033
						cg14067873	TSS200	25.7 ± 2.0	32.2 ± 2.1	6.5	0.026
						cg06376520	5′UTR;1stExon	25.5 ± 2.0	29.2 ± 1.8	3.6	0.044
						cg26207503	5′UTR;1stExon	25.9 ± 3.0	33.9 ± 2.5	8.0	0.049
						cg21126707	1stExon	28.7 ± 4.1	36.3 ± 3.8	7.6	0.033
						cg10868277	Body	29.7 ± 5.9	38.5 ± 5.6	8.8	0.035
3440095	*PSG3*	588.6 ± 427.3	67.6 ± 97.4	0.1	0.0017	cg07745725	TSS1500	48.9 ± 1.1	51.9 ± 1.6	2.9	0.038
						cg24313473	Body	14.0 ± 0.9	17.2 ± 1.5	3.3	0.044
4480292	*PSG5*	429.1 ± 283.6	60.7 ± 97.9	0.1	0.0017	cg01011943	5′UTR;1stExon	37.5 ± 1.8	41.3 ± 2.0	3.8	0.026
						cg01011943	5′UTR;1stExon	37.5 ± 1.8	41.3 ± 2.0	3.8	0.026
580711	*SMAD6*	725.9 ± 245.0	73.0 ± 39.3	0.1	0.0017	cg12216518	TSS1500;Body	20.4 ± 1.6	24.8 ± 2.0	4.4	0.035
						cg17860201	5′UTR;1stExon;Body	73.1 ± 1.5	78.1 ± 1.4	5.0	0.031
						cg27514333	Body	13.6 ± 1.8	17.5 ± 2.2	3.9	0.026
Upregulated											
5080132	*ACTA1*	391.8 ± 893.0	11408.8 ± 4221.9	29.1	0.0025	cg20025656	TSS1500	16.4 ± 1.1	20.8 ± 1.7	4.5	0.028
						cg02970384	Body	8.8 ± 0.8	12.0 ± 1.5	3.2	0.038
1230451	*TPM2*	147.9 ± 118.3	5491.1 ± 3076.8	37.1	0.0017	cg13969788	Body	25.0 ± 3.9	30.3 ± 4.1	5.3	0.031
						cg26607748	TSS200	8.7 ± 0.5	11.2 ± 0.9	2.5	0.044
4150379	*TNNT3*	32.5 ± 42.8	1258.1 ± 750.4	38.7	0.0017	cg26355894	TSS200	12.9 ± 1.8	16.5 ± 2.7	3.7	0.028
						cg15849872	5′UTR	11.3 ± 0.4	14.3 ± 0.9	3.0	0.028
						cg16911576	Body	73.3 ± 1.9	69.7 ± 2.2	−3.7	0.044
4760040	*NEB*	30.3 ± 33.5	1195.0 ± 957.8	39.4	0.0017	cg17254273	TSS1500	81.2 ± 1.2	77.9 ± 1.2	−3.3	0.041
3890743	*APOBEC2*	21.0 ± 23.0	989.5 ± 1067.7	47.0	0.0017	cg17548735	5′UTR;1stExon	24.9 ± 2.5	28.9 ± 2.7	4.0	0.033
						cg22375610	1stExon	12.8 ± 1.4	16.6 ± 1.7	3.9	0.026
6840092	*MYLPF*	213.4 ± 492.6	11507.5 ± 4592.8	53.9	0.0017	cg08130222	TSS200	54.8 ± 3.3	50.3 ± 3.3	−4.5	0.044
						cg09762897	Body	10.3 ± 0.4	12.9 ± 1.0	2.6	0.049
1070541	*MYH3*	200.9 ± 412.0	11286.7 ± 4171.6	56.2	0.0017	cg06891639	TSS1500	63.1 ± 2.8	55.6 ± 3.0	−7.5	0.049
6900360	*MYH7*	43.8 ± 63.2	4070.3 ± 2705.0	93.0	0.0017	cg22963791	Body	10.8 ± 0.5	13.3 ± 1.0	2.5	0.049
6620035	*CASQ2*	43.8 ± 60.1	4288.1 ± 2746.6	97.8	0.0017	cg17903306	TSS200	70.0 ± 3.3	58.8 ± 3.3	−11.2	0.049
3610402	*SLN*	24.4 ± 8.9	2969.5 ± 3289.5	121.6	0.0025	cg09773458	TSS1500	28.8 ± 2.7	35.0 ± 1.8	6.3	0.035

Data are presented as mean ± SD

We then performed correlation analyses between expression and methylation of corresponding CpG sites for the 4285 genes that had significant differences in both (Additional file 4: Table S3). Here, we found 1236 and 998 correlations between expression and methylation in myoblasts and myotubes, respectively (Additional file 4: Table S3), which is more than expected by chance based on χ^2^ tests (688 correlations with *P* < 0.05).

Using a luciferase assay, we continued to functionally study if DNA methylation affects the transcriptional activity of a gene that exhibits both differential methylation and expression during myogenesis. *MCM10*, which encodes a maintenance protein involved in DNA replication, was selected for this experiment as it showed increased methylation of several sites in the promoter region together with decreased expression after differentiation (Fig. 3h). A CpG-free luciferase expression vector containing 2000 bp of the *MCM10* promoter was either methylated in vitro with *SssI* or mock-methylated and transfected into cells. In line with our myotube data, we found that methylation of the *MCM10* promoter resulted in decreased transcriptional activity (Fig. 3i).

### Functional follow-up experiments identified IL-32 as a novel regulator of myogenesis and insulin sensitivity

Although it is well established that some key proteins regulate the myogenesis in human muscle cells [4], we hypothesized that there may be some additional proteins, not yet discovered, affecting the differentiation of myoblasts into myotubes and consequently muscle cell function. Genes previously not known to affect myoblast differentiation and which showed both altered expression and DNA methylation during myogenesis were therefore selected for functional follow-up studies.

**Fig. 4:**
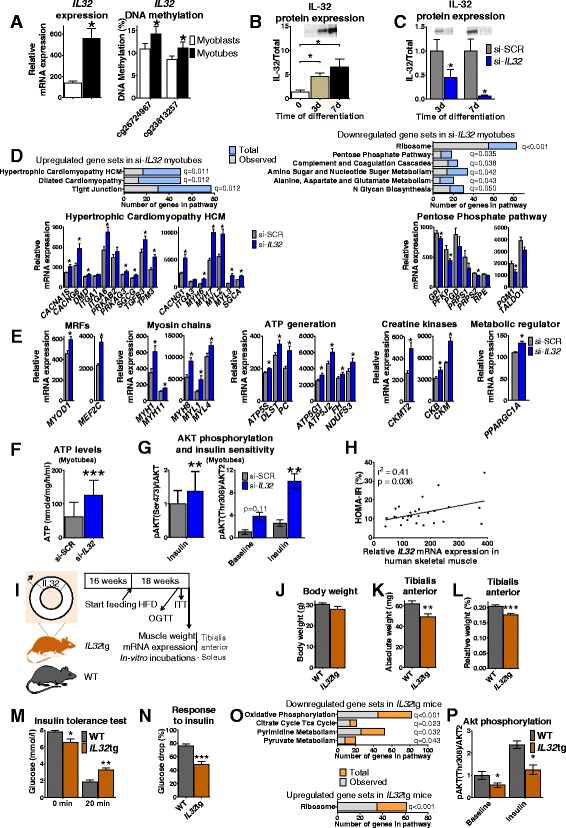
Silencing of *IL32* influences differentiation capacity and insulin signaling whereas *IL32* overexpression in mice impairs insulin sensitivity. **a** Array data of mRNA expression and DNA methylation (only significant sites) of *IL32* before versus after differentiation of primary human myoblasts from non-obese subjects (*n* = 13, *q < 0.05). **b** Protein expression of IL-32 in primary human myoblasts (0 h) and after 3 and 7 days of differentiation. Stain-free total protein staining was used for normalization. A representative blot is shown above the bars (*n* = 7). **c** Increased protein level of IL-32 found during differentiation was significantly blocked with siRNA after 3 and 7 days of differentiation of myoblasts. The average of Si-SCR is set to 1 at both time points. Stain-free total protein staining was used for normalization. Representative blots are shown above the bars. **d** Significantly enriched gene sets in Gene Set Enrichment Analysis (GSEA) based on differential gene expression in IL-32 deficient myotubes (day 7) versus control (si-SCR). Lower panels show expression of genes contributing to the gene sets Hypertrophic Cardiomyopathy HCM and Pentose Phosphate Pathway (*n* = 5, *q < 0.05). **e** A number of genes involved in myogenesis and metabolism with significantly different expression between IL-32 deficient myotubes (day 7) and control (*n* = 5, *q < 0.05). Silencing of *IL32* was associated with increased levels of ATP (**f**) and increased insulin-stimulated AKT phosphorylation at Ser473 and Thr308 (*n* = 3) (**g**) in differentiated myotubes (day 7). **h** The expression of *IL32* in skeletal muscle biopsies obtained from 27 adult men correlated positively with HOMA-IR (Pearson correlation). **i** Experimental set-up of the mouse study with *IL32*tg mice showing the duration of HFD, including time points for different analyses. **j** There was no difference in body weight between *IL32*tg and WT mice after 18 weeks on a HFD. *IL32*tg mice had significantly lighter tibialis anterior in absolute value (**k**) and in relation to body weight (**l**) compared to WT after 18 weeks on a HFD. *IL32*tg mice had lower glucose levels in the fasted state and higher levels 20 minutes after intravenous insulin administration compared with WT (**m**), resulting in a decreased insulin response (**n**). **o** Significantly enriched gene sets in GSEA based on differential gene expression in tibialis anterior from *IL32*tg versus WT mice after 18 weeks on a HFD (*n* = 6). **p** Soleus from *IL32*tg mice have decreased Akt phosphorylation at Thr308 after 30 minutes in vitro incubation with or without insulin (WT *n* = 4, *IL32*tg *n* = 5). Data are presented as mean ± SEM, *n* = 4 for cell experiments, *n* = 10 for WT mice, and *n* = 9 for *IL32*tg mice if nothing else stated. WT, Wild type mice (control); *IL32*tg, *IL32* transgenic mice; HFD, High fat diet; * *P* < 0.05, ** *P* < 0.01, *** *P* < 0.001 for figure **b** and **e**–**o**. Statistics were calculated with paired t-test for cell experiments and Mann–Whitney U test for mice data

The first selected candidate gene, *IL32* (encoding the cytokine Interleukin (IL)-32), showed a large increase in expression together with altered methylation in human myotubes compared with myoblasts (Fig. 4a).

IL-32 has previously been implicated in inflammatory diseases and cancer, but its role in myogenesis or muscle function remains unknown [34]. We next evaluated how the mRNA and protein expression patterns of IL-32 changed over time during differentiation of myoblasts into myotubes. Here, we found a significant increase in IL-32 expression 3 days after induction of differentiation and then a further increase after 7 days of differentiation (Fig. 4b and Additional file 1: Figure S2A). To assess whether IL-32 affects myogenesis or muscle function, we transfected primary human myoblasts with siRNA against *IL32* upon induction of differentiation. The cells were harvested at three time points – at 0 hours and 3 or 7 days after start of differentiation and siRNA transfection. An overview of the siRNA experiment is shown in Additional file 1: Figure S2B. Silencing of *IL32* resulted in significantly decreased IL-32 mRNA and protein levels both 3 and 7 days after transfection with siRNA (Fig. 4c and Additional file 1: Figure S2C).

We continued to investigate the effects of *IL32* silencing on gene expression using microarray analysis. We found 1231 unique genes that were differentially expressed between *IL32* silenced myotubes and cells treated with non-targeting siRNAs (si-SCR) (*q* < 0.05, Additional file 5: Table S4). We then applied GSEA and among the significant upregulated gene sets were several muscle-specific pathways (Fig. 4d and Additional file 5: Table S4). We also found increased expression of *MYOD1* and *MEF2C* as well as several MHCs and MLCs in *IL32* silenced cells (Fig. 4e), suggesting a negative role for IL-32 in myogenesis. Additionally, downregulated gene sets were related to amino acid and nucleotide turnover (Fig. 4d and Additional file 5: Table S4). Interestingly, a number of genes involved in ATP generation, e.g., in the citric acid cycle, the respiratory chain as well as creatine kinases were also upregulated, together with *PPARGC1A*, a transcriptional regulator of genes influencing energy metabolism (Fig. 4e).

As the microarray data indicated metabolic alterations in the *IL32* silenced cells, we proceeded to dissect the impact of IL-32 in myotubes. In line with our microarray data, we found increased ATP levels in *IL32* silenced cells (Fig. 4f). Moreover, IL-32-deficient myotubes exhibited increased insulin-stimulated AKT phosphorylation, as a measure of insulin sensitivity, at serine 473 (Ser473) and threonine 308 (Thr308) compared to control cells (si-SCR) (Fig. 4g). There was also a trend towards increased AKT phosphorylation at Thr308 at baseline (*P* = 0.11) (Fig. 4g). However, we could not detect any differences in glycogen synthase or TBC1D4 phosphorylation levels (data not shown).

These in vitro data in myotubes propose that IL-32 may contribute to impaired insulin sensitivity and metabolism. We subsequently tested if *IL32* expression in human skeletal muscle biopsies correlates with measures of insulin sensitivity analyzed in vivo using HOMA-IR in a cohort previously described [17]. Interestingly, *IL32* expression in muscle biopsies taken from middle-aged healthy men correlated positively with HOMA-IR (Fasting glucose (mmol/L) × Fasting insulin (mU/L)/22.5), further supporting a negative impact of IL-32 on insulin sensitivity (Fig. 4h).

To better understand the function of IL-32 on muscle growth and insulin sensitivity in vivo, we used transgenic mice expressing human *IL32* in skeletal muscle as well as other tissues [35]. *IL32* transgenic (*IL32*tg) and control (WT) mice were fed a Paigen high fat diet (HFD) [36] or control diet for 18 weeks, after which tibialis anterior and soleus muscle were excised; Fig. 4i shows the design of the mouse study. Despite similar body weight, *IL32*tg mice had significantly lighter tibialis muscle than WT after 18 weeks on HFD, both in absolute and relative terms (Fig. 4j–l). Moreover, *IL32*tg mice on HFD had significantly higher glucose levels and responded less well to insulin after a 20 minutes insulin tolerance test versus WT (Fig. 4m, n). On the other hand, their fasting glucose levels were reduced. No differences in muscle weight or insulin sensitivity were observed for mice on control diet (data not shown), nor were any differences in glucose levels observed during an oral glucose tolerance test or in food intake in any of the groups (data not shown).

We proceeded to investigate gene expression in tibialis anterior from *IL32*tg and WT mice on a HFD by microarray. We found 1821 unique genes that were differentially expressed with *P* < 0.05, but none with *q* < 0.05 (Additional file 5: Table S4), including *MYH2*, *TNNT3*, and *CKMT2*. GSEA revealed oxidative phosphorylation, citric acid cycle and pyruvate metabolism among significantly downregulated gene sets in *IL32*tg mice (Fig. 4o and Additional file 5: Table S4), which is in line with human myotube data where *IL32*-deficient cells had increased OXPHOS gene expression and ATP levels. Also in line with human myotube data, ribosome was the only upregulated gene set in *IL32*tg mice (Fig. 4o and Additional file 5: Table S4).

To further follow-up our AKT findings in human myotubes and the impaired insulin sensitivity in *IL32*tg mice, fresh soleus muscles from mice in each group were incubated in vitro for 30 minutes with and without insulin. *IL32*tg mice on HFD showed decreased Akt phosphorylation at Thr308 both with or without insulin stimulation (Fig. 4p), which is in accordance with our human myotube data (Fig. 4g). However, we could not detect any differences in Akt phosphorylation at Ser473 or ATP levels in soleus between the groups (data not shown).

In summary, these in vitro and in vivo experiments from both human and mouse support IL-32 as a novel player affecting myogenesis and muscle function.

Another novel candidate gene, *ARPP21* (encoding cAMP-regulated phosphoprotein), was selected for functional follow-up experiments based on its massive upregulation and altered methylation in myotubes compared to myoblasts (Additional file 1: Figure S2D). *ARPP21* showed a modest but significant increase in expression 3 days after induction of differentiation and then a dramatic increase after 7 days of differentiation (Additional file 1: Figure S2E). To study the influence of ARPP-21 on myogenesis, we used the same siRNA silencing set-up as for *IL32* but with fewer read-outs as presented in Additional file 1: Figure S2B. Silencing of *ARPP21* reduced *MYOG* expression at day 3 of differentiation and reduced *TNNI1* (encoding Troponin I Type 1) expression levels both at days 3 and 7 (Additional file 1: Figure S2F-G), proposing that the increased *ARPP21* expression during late myogenesis may function to stimulate structural proteins like troponin.

Two additional candidate genes, *SMAD6* and *PLAC8*, were selected for follow-up experiments based on their massive downregulation and altered methylation during myogenesis (Additional file 1: Figure S2H, I). Here, we used siRNA to study whether reduced expression of SMAD6 and PLAC8 in myoblasts affects proliferation, initiation of myogenesis, and/or myotube function (Additional file 1: Figure S2J). However, silencing the expression of these genes in our primary muscle cells only affected a few studied phenotypes (Additional file 1: Figure S2K, L).

### Impact of obesity on the DNA methylation pattern during differentiation of primary human myoblasts into myotubes

Although recent studies suggest that obesity impairs myogenesis in rodents, there is limited data from myoblast differentiation in obese humans [37]. We therefore investigated whether obesity affects the DNA methylation pattern during differentiation of primary human myoblasts into myotubes. The DNA methylation data described above in healthy non-obese subjects were compared with data from myoblasts and differentiated myotubes from 14 obese subjects (BMI > 30 kg/m^2^; Fig. 1a). The clinical characteristics of these subjects are presented in Table 1. Obese subjects had increased fat mass and insulin resistance estimated by HOMA-IR, as well as decreased HDL levels and VO_2max_, demonstrating a marked lower aerobic fitness level, compared with healthy non-obese subjects.

The average degree of DNA methylation was not significantly different between obese and non-obese subjects, neither in myoblasts nor in myotubes. Accordingly, the global methylation level increased in the same gene and CpG island regions in both obese and non-obese subjects after differentiation (Additional file 1: Figure S3A). However, among obese subjects, we identified 147,161 individual CpG sites with significantly altered DNA methylation in myoblasts versus myotubes (q < 0.05, Additional file 6: Table S5), which is approximately 3.7 times more than for non-obese subjects (*P*_χ2_ < 0.001) where 39,572 CpG sites changed significantly during myogenesis (Fig. 1a). 103,829 (71%) of the significant sites in obese subjects had increased methylation and 43,332 (29%) had decreased methylation in myotubes compared with myoblasts (Additional file 1: Figure S3B).

**Fig. 5:**
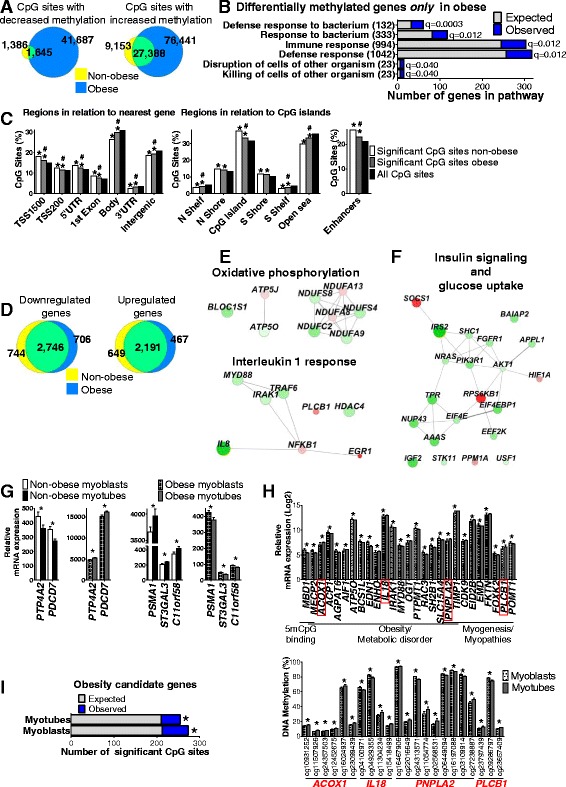
Differences in DNA methylation and gene expression before versus after differentiation of human primary myoblasts of obese and non-obese subjects. **a** Overlap of significant CpG sites with decreased and increased DNA methylation, respectively, after differentiation of human primary myoblasts of non-obese (*n* = 14) and obese (*n* = 14) subjects. **b** Significantly enriched gene ontology (GO)-terms (*q* < 0.05) of genes that exhibit differential DNA methylation only in obese. **c** Distribution of significant CpG sites that showed differential DNA methylation during myogenesis in non-obese and obese subjects compared with all analyzed CpG sites on the array and also between non-obese and obese in relation to gene regions, CpG island regions, and enhancer-regions. * *P*_χ2_ < 0.05 compared with all analyzed, # *P*_χ2_ < 0.05 between non-obese and obese subjects. **d** Overlap of downregulated and upregulated genes during myogenesis between non-obese (*n* = 13) and obese subjects (*n* = 13). Networks of certain genes only differentially expressed during myogenesis in obese (**e**) and non-obese (**f**) subjects, respectively, and related GO-terms to these genes. Green, downregulated; red, upregulated. Node size is based on expression fold change. **g** Genes (different probes) regulated in opposite directions during myogenesis in non-obese and obese subjects (* *q* < 0.05). **h** The top graph shows mRNA expression of genes only differentially expressed and methylated in obese subjects during myogenesis that are associated with DNA methylation, obesity, and/or metabolic disorders as well as myogenesis and/or myopathies. Values are presented as log2 to reduce magnitude and better visualize expression of all genes grouped in each category in one graph. The lower graph shows DNA methylation of significant CpG sites annotated to genes with a red box in the top graph (* *q* < 0.05). **i** Significant enrichment of the number of CpG sites within obesity candidate genes with differential DNA methylation at *P* < 0.05 between non-obese and obese subjects in myoblasts and myotubes, respectively (* *P*_χ2_ < 0.05). Data are presented as mean ± SEM

The overlap of significantly changed CpG sites between the obese and non-obese groups was 27,388 for sites with increased methylation and 1645 for sites with decreased methylation (Fig. 5a), revealing a large number of CpG sites uniquely methylated/demethylated in either of the groups during myogenesis (Additional file 7: Table S6). Significant pathways enriched for all differentially methylated genes in obese subjects are presented in Table 2.

We proceeded to identify unique genes where the methylation level only changed in obese or non-obese subjects during myogenesis. Here, one needs to take into account that different CpG sites in the same gene can change in the obese and/or non-obese subjects during differentiation, but this still represents the same gene in both groups. Thus, 5837 unique genes were differentially methylated during myogenesis only in the obese. Interestingly, these genes were significantly enriched in gene ontology (GO)-biological processes related to the immune response (Fig. 5b and Additional file 7: Table S6). The fact that epigenetic regulation is abnormal in cells from obese subjects, even after culturing them in the same condition as cells from non-obese subjects, demonstrates an altered epigenetic memory in muscle stem cells due to obesity. Methylation of 170 unique genes changed only in non-obese, but no enriched GO-biological process was found for these genes. To get a better view of where in the genome significant changes in methylation took place, the number of significant sites for each gene region was compared with the total number of analyzed sites for each region. In both obese and non-obese subjects, a higher proportion of significant sites than expected was observed in areas close to promoter regions, TSS, and CpG islands (*P*_χ2_ < 0.001; Fig. 5c). Nevertheless, obese subjects had a lower proportion than non-obese in TSS1500, TSS200, 5’UTR, 1^st^ exon, and CpG islands as well as a higher proportion in the gene body, 3’UTR, intergenic region, shelves, and open sea (*P*_χ2_ < 10^−11^). Additionally, 33,854 (23%) of the significant CpG sites in obese subjects were annotated to enhancer regions (Fig. 5c), which is significantly more than expected (*P*_χ2_ < 10^−104^). In non-obese subjects, there were 10,194 (26%) significant CpG sites annotated to enhancer regions, which is also more than expected (*P*_χ2_ < 10^−125^) and a higher proportion than in obese subjects (*P*_χ2_ < 10^−29^). The overrepresentation of significant CpG sites located in enhancer regions suggests that DNA methylation impacts on gene transcription during myogenesis.

Regarding non-CpG methylation in the obese, also here the number of significant sites was more than in non-obese subjects; 822 non-CpG sites (82%) had significantly changed methylation after differentiation (Additional file 6: Table S5).

### Impact of obesity on the transcriptome during differentiation of primary human myoblasts into myotubes

We proceeded to investigate if obesity affects the gene expression pattern during differentiation of primary human myoblasts into myotubes. Here, we compared the gene expression data described above in non-obese subjects with that of myoblasts and differentiated myotubes from 13 obese subjects (BMI > 30 kg/m^2^). The expression level of 7362 transcripts corresponding to 6080 unique genes changed significantly before versus after differentiation in obese subjects (Additional file 8: Table S7), which is slightly less than in non-obese subjects (Fig. 1a). 40% of the significantly changed transcripts in obese subjects were upregulated and 60% were downregulated. Of note, all identified MRFs, MLCs, and MHCs with differential expression in non-obese subjects also changed in the same direction in the obese (Fig. 2a, e and Additional file 8: Table S7), except for *MYL5*, which was significantly upregulated only in non-obese subjects.

GSEA of differentially expressed genes in obese subjects resulted in 15 downregulated gene sets and 17 upregulated gene sets (Additional file 8: Table S7). While all enriched gene sets for obese were also enriched for non-obese subjects, a number of upregulated gene sets including tight junctions, calcium signaling, and metabolism of various fatty acids as well as two downregulated gene sets – galactose metabolism and drug metabolism, other enzymes ﻿– were only enriched in the non-obese (Fig. 2b, c and Additional file 3: Table S2), suggesting that partial loss of muscle cell function occurs in obese humans.

Interestingly, we identified numerous unique changes in expression during myogenesis between obese and non-obese subjects, i.e., 1151 genes were only differentially expressed in obese and not in non-obese subjects, while 1376 genes only changed expression in the non-obese (Fig. 5d and Additional file 9: Table S8). Additionally, 2746 downregulated genes and 2191 upregulated genes after differentiation were overlapping between obese and non-obese subjects.

We next used GeneMANIA Cytoscape [38, 39] to visualize enriched biological processes associated with genes that only changed expression in either obese or non-obese subjects. Several GO-terms related to oxidative phosphorylation and an IL-1 response were significant only for obese subjects (Fig. 5e and Additional file 9: Table S8), whereas many GO-terms related to insulin signaling and glucose homeostasis were significant only for non-obese subjects (Fig. 5f and Additional file 9: Table S8). In contrast, GO-terms related to, e.g., the cell cycle, antigen recognition, and protein turnover were significant for both the unique genes found in either the obese or non-obese (Additional file 9: Table S8), suggesting a potential compensatory mechanism between genes uniquely regulated in the two groups. Interestingly, we also identified two genes, *PTP4A2* and *PDCD7*, which were significantly upregulated in the obese, while significantly downregulated in the non-obese during myogenesis (Fig. 5g). In contrast, *PSMA1*, *ST3GAL3*, and *C11orf58* (*SMAP*) were downregulated in obese while upregulated in non-obese subjects (Fig. 5g). Collectively, these data support metabolic and inflammatory disparities between differentiating muscle stem cells isolated from obese and non-obese subjects.

### Overlapping differences in DNA methylation and mRNA expression during differentiation in obese versus non-obese subjects

In obese subjects, 5197 genes showed both differential expression and differential DNA methylation of one or more CpG site(s) in myoblasts compared with myotubes (Table 4 and Additional file 10: Table S9). Interestingly, 220 of these genes did not show differential expression nor methylation in non-obese subjects and have been implicated in myopathies, epigenetic regulation, and metabolic conditions such as obesity and cardiovascular disease (Additional file 11: Table S10). Some of the genes, including *MECP2*, *ENHO*, *IL18*, and *PLCB1*, are presented in Fig. 5h. Furthermore, four genes (*MED10*, *NBPF11*, *PMAIP1*, and *ZFAND2A*) had differential expression together with differential methylation in non-obese subjects, but were not differentially expressed nor differentially methylated in obese subjects (Additional file 11: Table S10). These findings further emphasize an abnormal epigenetic regulation during differentiation of muscle stem cells from obese subjects potentially reflecting an impaired metabolism in vivo.

**Table 4:** Twenty genes with largest fold-change in expression that were also differentially methylated at one or more CpG site before versus after differentiation in obese

Expression						DNA methylation (%)					
IlluminaID	Gene symbol	Myoblasts	Myotubes	Ratio	*q* value	Probe ID	Gene region	Myoblasts	Myotubes	Difference	*q* value
Downregulated											
6840609	*CGB1*	1937.2 ± 3035.4	29.6 ± 10.0	0.02	0.0018	cg19220091	TSS1500	62.2 ± 1.8	59.7 ± 2.5	−2.5	0.011
						cg03671148	TSS1500	56.3 ± 2.2	54.5 ± 1.8	−1.8	0.032
						cg02355049	5′UTR;1stExon	36.9 ± 5.3	38.5 ± 6.2	1.6	0.044
						cg21042333	3′UTR	74.3 ± 5.4	71.5 ± 5.7	−2.8	0.0037
3710161	*LHB*	674.2 ± 1463.7	15.5 ± 6.1	0.02	0.0018	cg06452915	TSS1500	8.5 ± 1.4	11.3 ± 1.9	2.8	0.0029
						cg20173259	TSS1500	10.7 ± 1.9	14.3 ± 3.0	3.6	0.0029
						cg15504677	TSS1500	4.8 ± 0.4	4.4 ± 0.6	−0.4	0.023
130093	*MT1H*	1188.9 ± 1430.9	23.0 ± 9.7	0.02	0.0018	cg01507019	TSS1500	87.0 ± 4.3	85.5 ± 5.2	−1.5	0.037
						cg10014293	Body	32.3 ± 6.2	35.2 ± 6.3	2.9	0.037
						cg26937772	3′UTR	28.2 ± 14.2	34.9 ± 13.9	6.6	0.0029
1170300	*MT1G*	5699.4 ± 4458.8	183.9 ± 69.6	0.03	0.0018	cg01507019	TSS1500	87.0 ± 4.3	85.5 ± 5.2	−1.5	0.037
						cg07602841	TSS200	15.6 ± 3.7	19.6 ± 3.6	3.9	0.0037
						cg07791866	TSS200	9.2 ± 4.5	10.4 ± 5.1	1.1	0.0087
						cg02803037	TSS200	10.3 ± 5.6	11.9 ± 7.1	1.6	0.037
						cg16452857	TSS200	11.5 ± 2.7	10.5 ± 2.6	−1.1	0.037
4200372	*CGB5*	484.7 ± 969.2	17.6 ± 7.8	0.04	0.0018	cg04403474	TSS1500	14.6 ± 3.8	18.7 ± 5.8	4.1	0.0037
						cg24908058	5′UTR;1stExon	47.4 ± 6.3	49.1 ± 6.2	1.7	0.0044
						cg17497187	Body	68.6 ± 2.9	66.0 ± 2.9	−2.6	0.0070
670386	*ID1*	3065.6 ± 892.7	111.8 ± 80.4	0.04	0.0018	cg24056028	TSS1500	5.9 ± 0.7	7.1 ± 1.1	1.2	0.0087
						cg11815991	5′UTR;1stExon	6.4 ± 0.9	8.7 ± 0.9	2.3	0.0029
						cg27562717	5′UTR;1stExon	6.5 ± 0.8	8.3 ± 0.8	1.9	0.0029
						cg03154513	1stExon	6.6 ± 0.7	7.6 ± 0.9	1.0	0.032
1400634	*MT1M*	1632.7 ± 1996.4	73.5 ± 35.3	0.05	0.0018	cg05581701	TSS1500	14.6 ± 3.5	15.7 ± 3.6	1.2	0.032
						cg10638827	TSS1500	21.1 ± 2.8	24.7 ± 4.5	3.6	0.037
						cg02160530	TSS200	8.2 ± 0.9	10.4 ± 1.8	2.2	0.0044
						cg04994964	5′UTR;1stExon	12.8 ± 1.4	14.4 ± 2.2	1.7	0.032
						cg04523867	Body	57.5 ± 7.1	60.3 ± 6.0	2.7	0.044
4880609	*DKK1*	645.4 ± 526.5	61.6 ± 55.1	0.1	0.0018	cg25158147	TSS1500	13.9 ± 3.8	15.7 ± 3.2	1.8	0.032
						cg02162906	TSS1500	12.5 ± 1.8	14.1 ± 1.5	1.7	0.037
						cg02493604	TSS1500	11.3 ± 2.7	13.7 ± 3.7	2.4	0.037
						cg12621514	TSS1500	25.9 ± 7.7	32.1 ± 8.0	6.2	0.0029
						cg02302582	TSS1500	14.5 ± 2.1	17.4 ± 2.0	2.9	0.0044
						cg11988964	TSS1500	13.4 ± 2.5	16.6 ± 2.1	3.1	0.013
						cg25454948	TSS1500	11.7 ± 1.3	13.7 ± 1.3	2.0	0.013
						cg11931116	5′UTR;1stExon	12.8 ± 1.0	14.1 ± 0.9	1.3	0.016
						cg07684796	1stExon	7.0 ± 0.7	8.4 ± 1.4	1.4	0.023
						cg25751172	Body	16.4 ± 8.9	20.3 ± 9.9	3.9	0.0037
						cg01160882	Body	14.5 ± 4.5	17.3 ± 5.8	2.8	0.0058
						cg18956393	Body	8.2 ± 1.5	10.4 ± 3.3	2.2	0.0058
2140682	*MT1E*	263.1 ± 228.6	21.1 ± 6.6	0.1	0.0018	cg15134649	TSS1500	5.7 ± 0.9	6.5 ± 0.8	0.8	0.044
						cg07912888	TSS1500	26.4 ± 9.1	29.6 ± 10.3	3.2	0.011
						cg00178359	TSS200	22.6 ± 3.6	24.4 ± 3.4	1.8	0.027
						cg05743734	5′UTR;1stExon	5.9 ± 0.6	7.0 ± 0.9	1.1	0.0070
						cg20083730	5′UTR;1stExon	5.4 ± 0.7	6.3 ± 0.6	0.9	0.0087
						cg06463589	5′UTR;1stExon	5.0 ± 0.4	5.7 ± 1.0	0.7	0.016
						cg04793813	Body	6.7 ± 0.5	7.5 ± 0.8	0.8	0.032
2480452	*MYF5*	2049.5 ± 1210.7	213.3 ± 113.5	0.1	0.0018	cg04524478	TSS1500	31.2 ± 12.8	42.7 ± 11.6	11.5	0.0029
						cg10513852	TSS1500	19.0 ± 5.2	26.8 ± 10.9	7.8	0.0029
						cg14952949	TSS1500	16.4 ± 6.9	24.8 ± 10.2	8.4	0.0037
						cg14067873	TSS200	24.4 ± 5.2	30.3 ± 7.5	6.0	0.011
						cg26207503	5′UTR;1stExon	20.5 ± 7.0	27.5 ± 7.2	7.0	0.0029
						cg06376520	5′UTR;1stExon	22.4 ± 3.8	26.8 ± 6.0	4.4	0.013
						cg26998717	1stExon	12.5 ± 3.6	17.2 ± 3.5	4.8	0.0029
						cg21126707	1stExon	21.2 ± 8.9	28.7 ± 9.9	7.4	0.0044
						cg10868277	Body	21.4 ± 14.8	28.8 ± 15.3	7.5	0.0037
Upregulated											
1030431	*ACSL1*	204.5 ± 50.9	604.5 ± 165.3	3.0	0.0018	cg21390575	TSS1500	10.2 ± 2.3	12.3 ± 3.2	2.0	0.0029
						cg24170415	TSS1500	9.3 ± 1.1	11.5 ± 1.4	2.2	0.0029
						cg19487718	TSS1500	9.3 ± 2.3	11.0 ± 2.9	1.7	0.0070
						cg23684603	TSS200	9.5 ± 1.4	11.0 ± 1.9	1.5	0.044
						cg07619799	TSS200	6.7 ± 1.3	8.6 ± 1.2	1.9	0.0070
						cg07730946	5′UTR	73.8 ± 2.5	71.5 ± 3.2	−2.3	0.019
						cg11168687	5′UTR	39.2 ± 9.9	44.8 ± 8.9	5.7	0.019
						cg20823481	5′UTR	10.7 ± 1.9	13.1 ± 3.5	2.3	0.019
						cg20410192	5′UTR	7.1 ± 0.9	8.5 ± 1.0	1.5	0.032
						cg24721647	5′UTR	23.0 ± 16.2	28.7 ± 14.2	5.7	0.032
						cg00287477	Body	17.4 ± 5.4	22.7 ± 7.8	5.3	0.013
						cg03498175	Body	93.8 ± 1.3	92.6 ± 1.2	−1.2	0.019
						cg03422749	3′UTR	18.5 ± 3.7	23.1 ± 5.2	4.6	0.0044
7550301	*SLC8A3*	59.3 ± 23.1	250.8 ± 177.0	4.2	0.0018	cg13550670	TSS1500;Body	24.2 ± 8.1	28.4 ± 9.5	4.2	0.0058
						cg26033932	TSS1500;Body	40.7 ± 6.1	45.0 ± 8.2	4.3	0.0058
						cg21512124	TSS200	14.7 ± 1.6	16.6 ± 1.9	1.9	0.0087
						cg14174099	TSS200	5.5 ± 0.6	6.1 ± 0.7	0.5	0.037
						cg22823236	5′UTR	75.1 ± 6.1	68.9 ± 7.3	−6.3	0.0087
						cg00890485	5′UTR	12.6 ± 2.1	13.7 ± 1.8	1.1	0.044
						cg18296189	Body	10.1 ± 2.1	12.8 ± 2.9	2.7	0.011
						cg15823642	Body	33.0 ± 10.3	36.0 ± 9.7	3.0	0.0058
6840056	*MYL6B*	2410.8 ± 764.6	10375.0 ± 3226.6	4.3	0.0018	cg25012219	TSS1500	8.3 ± 1.1	9.9 ± 1.4	1.6	0.0070
						cg16017677	TSS1500;3′UTR	7.2 ± 1.0	8.9 ± 1.4	1.7	0.0029
						cg06400595	TSS1500;3′UTR	5.0 ± 0.7	5.7 ± 0.8	0.6	0.019
						cg27207452	TSS1500;3′UTR	6.9 ± 0.8	7.8 ± 1.0	0.9	0.023
						cg18176406	TSS200	7.5 ± 0.9	9.3 ± 1.0	1.8	0.0029
						cg20376421	TSS200	9.3 ± 2.5	11.4 ± 2.6	2.1	0.0029
						cg23309825	5′UTR	6.9 ± 0.7	8.4 ± 1.5	1.5	0.011
						cg25954162	5′UTR	10.9 ± 2.5	12.6 ± 3.9	1.7	0.027
1500349	*MYH6*	32.5 ± 18.7	467.9 ± 424.2	14.4	0.0018	cg26270038	TSS1500	55.4 ± 7.7	49.8 ± 6.3	−5.6	0.0044
						cg26083141	TSS1500	78.2 ± 5.5	75.6 ± 4.2	−2.6	0.013
						cg16803085	Body	83.6 ± 9.3	81.4 ± 8.8	−2.2	0.032
						cg21051473	Body	63.6 ± 8.2	61.0 ± 8.7	−2.6	0.037
						cg10949499	Body	64.1 ± 6.4	59.9 ± 5.9	−4.3	0.0044
						cg01593969	3′UTR	19.0 ± 10.2	22.2 ± 10.5	3.2	0.011
1010093	*SMPX*	26.6 ± 13.8	433.5 ± 274.3	16.3	0.0018	cg04489366	TSS1500	44.3 ± 16.3	49.5 ± 15.8	5.2	0.0087
						cg13118590	TSS1500	79.5 ± 6.7	75.9 ± 9.6	−3.5	0.027
						cg16168153	TSS200	73.2 ± 7.3	70.4 ± 8.6	−2.8	0.013
						cg00855923	TSS200	63.6 ± 12.5	58.6 ± 12.6	−5.0	0.019
7610451	*MYH2*	46.0 ± 39.8	1113.7 ± 1154.5	24.2	0.0018	cg18432105	TSS1500	75.6 ± 10.6	68.1 ± 11.9	−7.5	0.0029
						cg27569752	TSS200;5′UTR;1stExon	20.9 ± 8.4	24.8 ± 8.6	3.9	0.0070
						cg06220958	5′UTR	15.9 ± 4.5	19.5 ± 7.0	3.6	0.011
						cg07416502	Body	81.4 ± 4.6	77.6 ± 5.3	−3.9	0.011
4730435	*MYL1*	208.3 ± 316.3	5624.2 ± 4526.8	27.0	0.0018	cg27305342	TSS1500;Body	76.8 ± 9.5	73.4 ± 9.1	−3.4	0.027
						cg21468315	TSS200	74.8 ± 7.5	70.3 ± 8.0	−4.5	0.016
						cg10727812	1stExon	86.5 ± 6.6	83.5 ± 4.2	−3.0	0.037
						cg11059341	Body	72.4 ± 8.4	67.6 ± 6.5	−4.8	0.044
1070541	*MYH3*	373.1 ± 523.9	10804.2 ± 3658.5	29.0	0.0018	cg11887833	TSS1500	88.9 ± 7.5	85.7 ± 7.5	−3.1	0.0029
						cg22108292	TSS1500	74.0 ± 9.4	67.2 ± 10.5	−6.8	0.0070
						cg06891639	TSS1500	63.2 ± 9.6	57.7 ± 8.3	−5.5	0.016
						cg17944891	TSS1500	69.8 ± 6.7	64.2 ± 8.1	−5.5	0.016
						cg21430748	Body	76.3 ± 4.8	73.6 ± 5.2	−2.8	0.0070
6900360	*MYH7*	92.3 ± 128.0	4031.0 ± 2324.9	43.7	0.0018	cg26670875	TSS1500	27.7 ± 7.2	33.6 ± 5.8	5.9	0.0044
						cg01203550	TSS200	19.6 ± 9.1	22.1 ± 9.3	2.5	0.011
						cg08240074	5′UTR	73.3 ± 10.2	67.1 ± 10.0	−6.2	0.0037
						cg14030388	Body	81.9 ± 3.6	78.9 ± 3.4	−2.9	0.0058
						cg23123918	Body	77.0 ± 6.1	73.9 ± 6.0	−3.1	0.0087
						cg22963791	Body	9.7 ± 1.2	11.4 ± 2.1	1.7	0.011
						cg08209104	Body	45.6 ± 6.4	43.4 ± 6.9	−2.2	0.013
						cg21176875	Body	74.0 ± 7.2	70.5 ± 6.3	−3.5	0.013
						cg13785779	Body	53.8 ± 9.8	50.3 ± 9.8	−3.5	0.027
						cg21242212	Body	60.1 ± 8.5	57.9 ± 7.4	−2.2	0.037
3610402	*SLN*	28.0 ± 13.3	2635.2 ± 3930.7	94.1	0.0018	cg09773458	TSS1500	26.4 ± 8.7	35.2 ± 11.1	8.7	0.0070
						cg05929864	TSS1500	35.8 ± 8.0	42.8 ± 6.4	7.0	0.0087
						cg17971003	TSS200	43.0 ± 12.1	49.1 ± 11.3	6.2	0.0087
						cg16476427	TSS200	16.5 ± 7.2	22.6 ± 10.4	6.0	0.011
						cg24307368	TSS200	9.7 ± 1.9	12.7 ± 5.0	3.0	0.016
						cg11117131	TSS200	23.3 ± 6.5	27.7 ± 8.5	4.3	0.032
						cg12237269	5′UTR;1stExon	82.9 ± 3.8	84.2 ± 4.0	1.3	0.037

Data are presented as mean ± SD

We then performed correlation analyses between the expression and methylation of corresponding CpG sites for the 5197 genes that had significant differences in both expression and methylation in obese subjects (Additional file 10: Table S9). Here, we found 3515 and 4014 correlations between expression and methylation in myoblasts and myotubes, respectively (Additional file 10: Table S9), which is more than expected by chance (2351 correlations with *P* < 0.05).

### Differential DNA methylation and gene expression in myoblast and/or myotubes between obese and non-obese subjects

We further tested if any individual CpG sites were differentially methylated when comparing myoblasts between obese and non-obese or when comparing myotubes between obese and non-obese, respectively. While 29,337 CpG sites were differentially methylated in myoblasts from obese versus non-obese at *P* < 0.05, only methylation of two CpG sites (near *ZNF629* and *SDCCAG1*) were significantly different based on FDR < 5% (Additional file 12: Table S11). In myotubes, 28,884 sites were differentially methylated between obese and non-obese subjects at *P* < 0.05, but none had FDR < 5%. Nevertheless, we proceeded to test if there was an over-representation of identified CpG sites with *P* < 0.05 in/near known candidate genes for obesity based on discoveries in genome-wide association studies (*P* < 1.0 × 10^−5^; www.genome.gov/gwastudies accessed 22 August 2012). Out of 3472 analyzed CpG sites annotated to 129 candidate genes for obesity, 273 CpG sites in 93 genes had differential methylation in obese versus non-obese at *P* < 0.05 in myoblasts and 253 CpG sites in 86 genes had *P* < 0.05 in myotubes (Fig. 5i and Additional file 12: Table S11). This is more than expected by chance (myoblasts; *P*_χ2_ = 2.3 × 10^−5^, and myotubes; *P*_χ2_ = 0.001), thus suggesting that epigenetic variation may influence the genetic risk for metabolic disorders [40]. We continued to compare the transcriptome profile in myoblasts and myotubes from obese versus non-obese subjects. After FDR correction, there were no significant differences in gene expression between obese and non-obese subjects in myoblasts nor in myotubes. Neither did we find any enrichment of candidate genes for obesity among the genes with altered expression at *P* < 0.05 between obese and non-obese subjects in myoblasts or myotubes.

### Enzymes regulating DNA methylation in obese and non-obese subjects

**Fig. 6:**
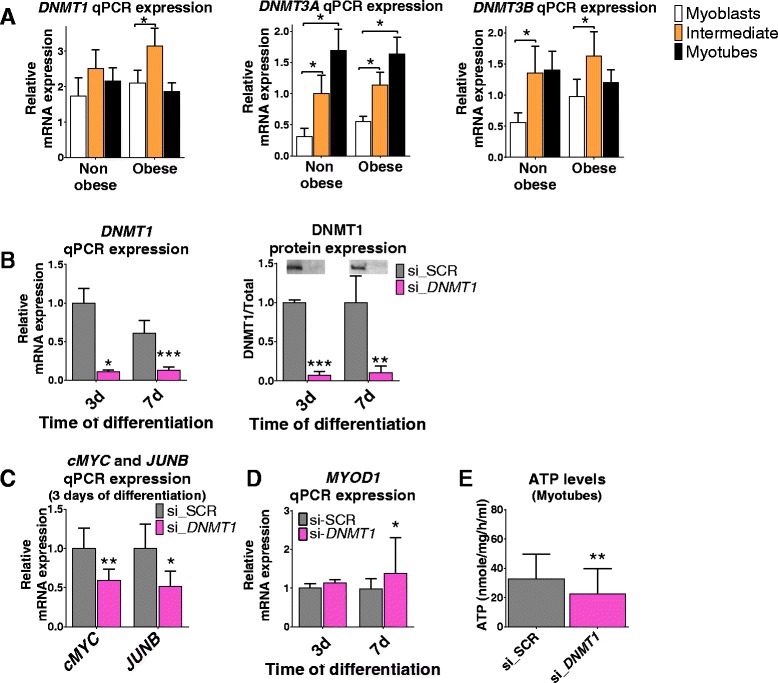
*DNMT1, DNMT3A*, and *DNMT3B* during differentiation of primary human myoblasts. **a** mRNA expression of *DNMT1, DNMT3A*, and *DNMT3B* in myoblasts, after 2 days of differentiation (intermediate) and in differentiated myotubes from six non-obese and six obese subjects. **b** siRNA silencing of *DNMT1* in the myoblasts was confirmed after 3 and 7 days of differentiation at mRNA and protein levels. The average of Si-SCR is set to 1 at both time points for protein expression. Stain-free total protein staining was used for normalization. Representative blots are shown above the bars. Silencing of *DNMT1* resulted in reduced expression of *cMYC* and *JUNB* after 3 days of differentiation (**c**) as well as increased expression of *MYOD1* (*n* = 7) (**d**) and reduced levels of ATP (*n* = 3) in myotubes (day 7) (**e**). Data are presented as mean ± SEM, *n* = 4 for (**b**–**e**) if nothing else stated, except protein levels in (**b**) at day 3, where *n* = 3. *P < 0.05, **P < 0.01, ***P < 0.001

To further assess why methylation of approximately 3.7 times more sites changed in obese compared with non-obese subjects during differentiation, we analyzed the expression of all three catalytically active DNA methyltransferases (DNMTs) with qPCR at three different time points, in myoblasts and after 2 (intermediate) and 7 (myotubes) days of differentiation (Fig. 6a). Interestingly, *DNMT1* expression was only significantly upregulated in obese, but not in non-obese subjects, after 2 days of differentiation. The significant changes in the expression patterns for *DNMT3A* and *DNMT3B* were similar in obese and non-obese subjects (Fig. 6a).

We next silenced *DNMT1* to further study its role during myogenesis (Additional file 1: Figure S2B) and reduced mRNA and protein levels were achieved after 3 and 7 days of differentiation (Fig. 6b). This resulted in reduced expression of two cell cycle regulators, *cMYC* and *JUNB*, after 3 days, as well as increased expression of the myogenic marker *MYOD1* and decreased ATP levels after 7 days, of differentiation (Fig. 6c–e). Hence, DNMT1 seems to affect myogenesis and cell function.

Differences in DNA methylation could potentially also be explained by differences in the availability of methyl groups or other epigenetic modifiers. Notably, the expression pattern of 10 enzymes and modifiers involved in the one carbon metabolism and generation of the methyl donor S-adenosylmethionine changed significantly in either the obese and non-obese during differentiation (Additional file 1: Figure S3C). These differences may also contribute to the abnormal methylation changes seen in the obese, i.e., that approximately 3.7 times more CpG sites changed in obese compared with non-obese subjects during myogenesis.

### Secretion of cytokines and growth factors from myoblasts and myotubes of obese and non-obese subjects

Extracellular stimuli, like circulating cytokines and growth factors, influence myogenesis [4], and their secretion may be altered by obesity. We therefore analyzed the secretion of CCL2, TGF-β3, IL-32, IL-6, and TNF-α, all previously implicated in myogenesis (except IL-32) [41], in the culture media from myoblasts and myotubes of obese and non-obese subjects.

As expected, the secretion of CCL2 increased after differentiation in non-obese and correlated with its corresponding mRNA level in myoblasts (Additional file 1: Figure S3D). The same secretion pattern was seen for the obese. TGF-β3 was the cytokine with highest fold-change in mRNA expression between myoblasts and myotubes in both obese and non-obese subjects (Additional file 1: Figure S3E). However, significantly increased TGF-β3 secretion after differentiation was only observed in the obese (Additional file 1: Figure S3E). Thereby, we also identified a secretory discrepancy between myotubes generated from muscle stem cells of obese and non-obese subjects, although cultured under the same condition. IL-32 could not be detected in culture media from myoblasts (Additional file 1: Figure S3F), which is in agreement with the IL-32 data presented above (Fig. 4a, b). However, we could detect IL-32 secretion from myotubes from 46% of the non-obese and 20% of the obese subjects, suggesting that IL-32 may be a myokine. However, there was no significant difference in secretion levels between the obese and non-obese (Additional file 1: Figure S3F). No differences in secretion of IL-6 and TNF-α were detected (data not shown).

### Technical and biological validation

We finally tested if our microarray data could be technically and biologically validated. For technical validation, we compared our mRNA microarray data with qPCR data generated from the same samples for *DNMT1* and found significant correlations between the microarray and qPCR data (r = 0.57, *P* = 0.002). For biological validation, we compared our microarray data for *IL32* and *ARPP21* with qPCR data generated in a different set of cells cultured at different time points. Importantly, we found the same changes in the expression pattern for these two genes in cell culture experiments performed at different time points (Fig. 4a, Additional file 1: Figure S2A and D–E).

## Discussion

This study highlights the importance of genome-wide epigenetic changes during human myogenesis. We found numerous epigenetic and transcriptional alterations during differentiation of primary human muscle stem cells. Importantly, we identified IL-32 as a novel factor influencing myogenesis and insulin sensitivity. Moreover, we observed abnormal epigenetic changes and gene expression patterns during differentiation of human muscle stem cells from obese compared with non-obese subjects.

Here, we present the first study analyzing the genome-wide DNA methylation and gene expression patterns during differentiation of activated primary muscle stem cells isolated from a large human cohort including both obese and non-obese subjects. We observed overwhelming de novo methylation during myogenesis, supporting DNA methylation as an important epigenetic mechanism coordinating cell-specific gene expression as the cells become more specialized. Importantly, myogenesis was accompanied by dynamic methylation and expression changes of *PAX7*, all major MRFs and MEF2s (*MYOD*, *MYF5*, *MYOG*, *MYF6*, *MEF2A*, *MEF2C*, M*EF2D*), the MHC and MLC families, as well as mitochondrial proteins, thereby demonstrating that the cells were successfully differentiated. Moreover, GSEA of genes differentially expressed following differentiation showed downregulation of gene sets involved in the cell cycle and DNA synthesis, reflecting that the myoblasts go into cell cycle arrest and start to differentiate. Upregulated gene sets reflect myofiber formation (e.g. hypertrophic cardiomyopathy, cardiac muscle contraction, and calcium signaling pathway) and support that the myotubes line up close to each other (extracellular matrix receptor interaction and cell adhesion), which can also be seen in the pictures of our differentiated myotubes. Interestingly, the GSEA revealed a major shift in metabolism and substrate choice, with the myoblasts having higher expression levels of gene sets regulating fructose, mannose and galactose metabolism, and the pentose phosphate pathway and the myotubes having higher expression levels of gene sets regulating fatty acid metabolism as well as tryptophan and linoleic acid metabolism. Altogether, this proposes a shift from using carbohydrates as fuels in dividing myoblasts towards using fatty acids and amino acids as fuels and building blocks in mature myotubes. This is in accordance with a recent study showing that activated satellite cells (myoblasts) rely on glycolysis [42]. Ryall et al. [42] further describe how a shift in metabolic substrate utilization, partly through histone modifications, regulates cell development and cell fate, a process termed ‘metabolic reprogramming’. In our study, numerous changes in DNA methylation induced by differentiation accompany the shift in expression of gene sets regulating metabolism, thereby suggesting a close interaction between epigenetic modifications and metabolism during myogenesis.

Interestingly, two pregnancy-associated gene families, PSGs and CGBs, were among the most downregulated gene groups characterizing the transition from myoblast to myotube. The PSGs are glycoproteins secreted by the placental trophoblasts into the maternal bloodstream during pregnancy and are associated with fetal well-being [29]. The CGBs are also secreted by the placental trophoblasts with the purpose of maintaining the corpus luteum during pregnancy [30]. Neither the PSGs nor CGBs have ever been detected or described in relation to skeletal muscle, myoblasts, or myogenesis. However, from our studies, it seems as if they could have a major impact on human myoblast regulation. Another pregnancy associated protein, leukemia inhibitory factor, which is normally also expressed by trophoblasts to facilitate implantation, has in two decades been known to stimulate proliferation of myoblasts [43], was tested for its effect of myoblast transplantation in animals [44], and has been shown to be secreted from myoblasts [45]. These findings indicate possible secretory similarity between trophoblasts and myoblasts. Another interesting gene group downregulated during myoblast differentiation is the metallothioneins, which also showed dynamic changes in DNA methylation during myogenesis. They have been sparsely described in relation to skeletal muscle, but appear to increase in atrophying muscles [46] and might be related to a high requirement of zinc for metabolic activities during the early stage of myoblast differentiation [47].

In addition to the identified novel gene groups described above, a number of unique genes displayed transcriptional and epigenetic changes, and could thus potentially be involved in regulating myogenesis and muscle function. We selected some of these novel candidate genes, including *IL32*, which was dramatically upregulated during differentiation, for functional follow-up experiments. By combining in vitro and in vivo experiments, IL-32 was found to have negative effects on both myogenesis and insulin sensitivity in mice and humans. Silencing *IL32* in human myotubes increased insulin-stimulated AKT phosphorylation and intracellular ATP levels. These cells also had increased expression of OXPHOS genes and *PPARGC1A*, which is in line with changes seen in muscle during exercise [48, 49]. To further explore this relationship in vivo, we studied mice overexpressing human *IL32* and fed a HFD. *IL32*tg mice responded less well to insulin, which resulted in higher glucose levels, had lower insulin-stimulated Akt phosphorylation in soleus, and decreased OXPHOS gene expression in tibialis anterior, supporting our in vitro data. We could not detect any alteration in ATP levels in soleus muscle from *IL32*tg mice, which may reflect that the phenotype is more pronounced in some muscle types or in pure muscle cells. Remarkably, we also found a negative association between muscle-derived *IL32* levels and whole body insulin sensitivity in humans in vivo. Our experiments further suggest that IL-32 has negative effects on myogenesis and muscle growth/maintenance by inhibiting expression of myogenic genes, potentially through reduced AKT signaling. For example, the expression of *MEF2C*, *MYOD1*, and *DMD* was higher in IL-32-deficient myotubes and *IL32*tg mice had decreased muscle mass. Upregulation of IL-32 during myogenesis may therefore function to inhibit muscle overgrowth. Even if muscle function and mass is of great importance for a good quality of life, the tissue is metabolically expensive. It may therefore have been evolutionary beneficial for survival to develop mechanisms that control muscle growth and reduce insulin sensitivity [50], while with the modern lifestyle it contributes to disease. Interestingly, several of the effects of IL-32 on muscle cells resemble those of myostatin, a master regulator of muscle growth. Myostatin is a myokine, which increases during myogenesis, signals through the TGF-β pathway, and regulates myoblast differentiation [51, 52]. Increased expression of myostatin is associated with muscle wasting, while its knockdown causes hypertrophy. One of the mechanisms by which myostatin exerts its function is through reduced AKT phosphorylation. It also seems to regulate glucose utilization and its expression is positively correlated with insulin resistance [53]. Myostatin inhibitors have been intensively studied as pharmaceutical targets for muscle atrophy and mice treated with anti-myostatin antibodies have increased muscle mass and improved muscle insulin sensitivity [54]. In conclusion, our findings suggest that IL-32 resembles myostatin in several ways, warranting future studies on whether IL-32 inhibitors may improve muscle mass and function. We also demonstrate, for the first time, that IL-32 is secreted from myotubes, further encouraging studies of its function in muscle biology.

DNA methylation was initially considered a silencing mark. However, more recent data show that the impact of methylation on expression varies with genomic context and methylation may both negatively and positively affect the transcriptional activity, regulating splicing and use of alternative promoters [33]. For example, the *IL32* promoter does not reside in a CpG island and increased methylation may therefore be observed together with increased expression. While large absolute changes in DNA methylation are observed in cancer cells, modest methylation changes are commonly seen in non-tumorigenic cells [17, 18, 55]. Nevertheless, the effects may still be of biological relevance, particularly in complex diseases where numerous changes with smaller effect sizes are known to affect disease susceptibility [56]. Here, we found both negative and positive correlations between expression and methylation for numerous genes in both myoblasts and myotubes, emphasizing the complexity of epigenetic regulation of expression as mentioned above. The luciferase assay further showed that increased promoter methylation directly influences the transcriptional activity of *MCM10*, whose expression and methylation changed during myogenesis.

Obesity is a complex, multifactorial disorder rapidly increasing worldwide. Although interactions between genetic and environmental factors are known to affect a person’s susceptibility to weight gain, identified genetic variants do only explain a modest proportion of the estimated heritability of obesity and related diseases [56]. This has resulted in a growing interest in understanding the role of epigenetics in the increasing prevalence of obesity. Indeed, obesity, overfeeding, physical activity, and genetic variation have been shown to alter the genome-wide DNA methylation pattern in human adult tissues [17, 18, 57–61]. However, current knowledge about the impact of obesity on the epigenetic pattern during differentiation of human stem cells remains scarce.

The novel observation of altered epigenetic and transcriptomic regulation during differentiation of myoblasts isolated from obese individuals shows that muscle stem cells are profoundly altered/programmed by the systemic nutritional environment and/or physical inactivity. Although the muscle stem cells from obese and non-obese subjects were cultured during identical conditions, the number of DNA methylation changes induced by differentiation was three-fold higher in the obese. This clearly demonstrates a different epigenetic reorganization during the transition from myoblast to myotube as a memory of the obese environment. DNMT1 is a methyltransferase known to maintain methylation during replication, but has also been found to affect de novo methylation during cell differentiation [62, 63]. Hence, the more active de novo methylation observed in cells from obese may be explained by the increased *DNMT1* expression in early myogenesis. We further silenced *DNMT1* throughout myogenesis and found decreased levels of *cMYC* and *JUNB* at day 3 of differentiation as well as increased *MYOD1* expression in differentiated myotubes, suggesting that DNMT1 may also affect the cell cycle and differentiation in these cells.

While approximately three times more methylation changes took place in the obese during differentiation, only a modest number of differences were observed in myoblasts from obese versus non-obese subjects. It may be explained by large variances within groups. Indeed, the phenotype of a complex disease is commonly caused by a combination of many small variations at numerous loci that require large cohorts for detection [64].

A surprisingly large number of genes were uniquely changed in expression either in obese cells (1151) or non-obese cells (1376) after differentiation, underscoring that both DNA methylation and gene expression changes vary genome-wide between the two cell populations. Interestingly, several genes that only showed expression changes in the non-obese are known to regulate insulin signaling and glucose homeostasis. Indeed, the obese subjects included in this study were insulin resistant and had lower oxidative capacity based on VO_2max_. Among the genes that showed both differential gene expression and methylation in only obese, were genes previously associated with dysfunctional myogenesis, e.g., *PLCB1* and *CDK9* [65, 66], genes linked to obesity or other metabolic diseases, including *IL18*, *PNPLA2*, and *ENHO* [67–69], as well as genes for which an association to obesity has never been described. The latter may be interesting targets to study further in relation to obesity and myogenesis. Of interest are also five genes that were regulated in opposite direction in obese compared with non-obese. One of these, *PSMA1* has previously been associated with fatty liver in obese subjects [70]. Additionally, *PTP4A2* was recently implicated in hematopoietic stem cell self-renewal [71] and *PDCD7* encodes a component of the spliceosome [72].

## Conclusions

This study provides a comprehensive map of the dynamic methylome and transcriptome during differentiation of human muscle stem cells and identified numerous of new myogenic targets. Importantly, we showed that IL-32 is a novel candidate influencing human myogenesis and insulin sensitivity both in vivo and in vitro. Our data further highlight that the metabolic environment influences the epigenetic memory in human muscle stem cells, thereby altering the transcriptome and epigenome during adult myogenesis in obese individuals.

## Additional files

Additional file 1: Supplemental methods. **Table S12.** Antibodies used for western blot. **Figure S1.** Characterization of the primary human myoblasts**. Figure S2.** Silencing of *ARPP21*, *SMAD6*, and *PLAC8* during differentiation of primary human myoblasts**. Figure S3.** Differences in methylation, transcription, and secreted cytokine levels before versus after differentiation of primary human myoblast from obese and non-obese subjects. (PDF 1126 kb)
Additional file 2: Table S1. CpG sites and non-CpG sites that exhibit differential DNA methylation (q < 0.05) before versus after differentiation of human primary myoblasts of 14 non-obese subjects, as well as enriched KEGG pathways (adjusted *P* < 0.05) of the genes annotated to these significant CpG sites (mean ± SD). (XLSX 3195 kb)
Additional file 3: Table S2. Differentially expressed genes (q < 0.05) before versus after differentiation of human primary myoblasts of 13 non-obese subjects, as well as significant results of gene set enrichment analysis (FDR adjusted *P* < 0.05) and JASPAR motif search (*P*_Bonferroni_ < 0.05) for these genes (mean ± SD). (XLSX 669 kb)
Additional file 4: Table S3. Genes with overlapping changes in gene expression and DNA methylation before versus after differentiation of human primary myoblasts of non-obese subjects, followed by correlation analyses between expression and methylation of corresponding CpG sites for genes that had significant differences in both (13,769 correlations in total in myoblasts and myotubes) (mean ± SD). (XLSX 1875 kb)
Additional file 5: Table S4. Differentially expressed genes (q < 0.05) between IL-32-deficient primary human myotubes and controls (n = 5) and significant results of Gene Set Enrichment Analysis (GSEA; FDR adjusted *P* < 0.05) for these genes, followed by differentially expressed genes (*P* < 0.05) between *IL32*tg and WT mice (n = 6) and significant results of GSEA (FDR adjusted *P* < 0.05) for these genes (mean ± SD). (XLSX 299 kb)
Additional file 6: Table S5. CpG sites and non-CpG sites that exhibit differential DNA methylation (q < 0.05) before versus after differentiation of human primary myoblasts of 14 obese subjects (mean ± SD). (XLSX 10313 kb)
Additional file 7: Table S6. CpG sites that exhibit differential DNA methylation (q < 0.05) during myogenesis in either non-obese or obese subjects, as well as enriched gene ontology-biological processes for genes differentially methylated at one or more CpG sites during myogenesis only in obese subjects (mean ± SD). (XLSX 8191 kb)
Additional file 8: Table S7. Differentially expressed genes (q < 0.05) before versus after differentiation of human primary myoblasts of 13 obese subjects, as well as significant results of Gene Set Enrichment Analysis (FDR adjusted *P* < 0.05) for these genes (mean ± SD). (XLSX 624 kb)
Additional file 9: Table S8. Genes differentially expressed (q < 0.05) during myogenesis in either non-obese or obese subjects, as well as gene ontology-terms enriched in GeneMANIA Cytoscape for these genes (*P* < 0.05) (mean ± SD). (XLSX 259 kb)
Additional file 10: Table S9. Genes with overlapping changes in gene expression and DNA methylation in human primary myoblasts versus myotubes of obese subjects followed by correlation analyses between expression and methylation of corresponding CpG sites for genes that had significant differences in both (47,011 correlations in total in myoblasts and myotubes) (mean ± SD). (XLSX 5784 kb)
Additional file 11: Table S10. Genes differentially expressed and methylated between myoblasts and myotubes (q < 0.05) in either non-obese or obese subjects (mean ± SD). (XLSX 121 kb)
Additional file 12: Table S11. CpG sites with differential DNA methylation between non-obese and obese subjects in myoblasts and myotubes, respectively (*P* < 0.05), as well as differentially methylated candidate genes for obesity between non-obese and obese subjects in myoblasts and myotubes, respectively (*P* < 0.05) (mean ± SD). (XLSX 4431 kb)

## Abbreviations

BMI: body mass index
CGB: chorionic gonadotropin beta polypeptides
DNMT: DNA methyltransferase
FDR: false discovery rate
GO: gene ontology
GSEA: gene set enrichment analysis
HFD: high fat diet
HOMA-IR: homeostasis model assessment-estimated insulin resistance
IL-32: interleukin 32
*IL32*tg: IL-32 transgenic
MEF2: myocyte enhancer factor-2
MHC: myosin heavy chain
miRNA: microRNA
MLC: myosin light chain
MRF: myogenic regulatory factor
OXPHOS: oxidative phosphorylation
PSG: pregnancy-specific beta-1-glycoproteins
TSS: transcription start site
UTR: untranslated region
WT: wild type

## References

[1] Serrano AL, Baeza-Raja B, Perdiguero E, Jardi M, Munoz-Cinoves P (2008). Interleukin-6 is an essential regulator of satellite cell-mediated skeletal muscle hypertrophy. Cell Metab.

[2] Petrella JK, Kim JS, Mayhew DL, Cross JM, Bamman MM (2008). Potent myofiber hypertrophy during resistance training in humans is associated with satellite cell-mediated myonuclear addition: a cluster analysis. J Appl Physiol.

[3] Relaix F, Zammit PS (2012). Satellite cells are essential for skeletal muscle regeneration: the cell on the edge returns centre stage. Development.

[4] Ciciliot S, Schiaffino S (2010). Regeneration of mammalian skeletal muscle. Basic mechanisms and clinical implications. Curr Pharm Des.

[5] Liu N, Nelson BR, Bezprozvannaya S, Shelton JM, Richardson JA, Bassel-Duby R, Olson EN (2014). Requirement of MEF2A, C, and D for skeletal muscle regeneration. Proc Natl Acad Sci U S A.

[6] Varley KE, Gertz J, Bowling KM, Parker SL, Reddy TE, Pauli-Behn F, Cross MK, Williams BA, Stamatoyannopoulos JA, Crawford GE (2013). Dynamic DNA methylation across diverse human cell lines and tissues. Genome Res.

[7] Tsumagari K, Baribault C, Terragni J, Varley KE, Gertz J, Pradhan S, Badoo M, Crain CM, Song L, Crawford GE (2013). Early de novo DNA methylation and prolonged demethylation in the muscle lineage. Epigenetics.

[8] Miyata K, Miyata T, Nakabayashi K, Okamura K, Naito M, Kawai T, Takada S, Kato K, Miyamoto S, Hata K (2015). DNA methylation analysis of human myoblasts during in vitro myogenic differentiation: de novo methylation of promoters of muscle-related genes and its involvement in transcriptional down-regulation. Hum Mol Genet.

[9] Edelstein SL, Knowler WC, Bain RP, Andres R, Barrett-Connor EL, Dowse GK, Haffner SM, Pettitt DJ, Sorkin JD, Muller DC (1997). Predictors of progression from impaired glucose tolerance to NIDDM: an analysis of six prospective studies. Diabetes.

[10] Thompson LV (2002). Skeletal muscle adaptations with age, inactivity, and therapeutic exercise. J Orthop Sport Phys.

[11] Fu X, Zhu M, Zhang S, Foretz M, Viollet B, Du M (2016). Obesity impairs skeletal muscle regeneration through inhibition of AMPK. Diabetes.

[12] Green CJ, Pedersen M, Pedersen BK, Scheele C (2011). Elevated NF-kappaB activation is conserved in human myocytes cultured from obese type 2 diabetic patients and attenuated by AMP-activated protein kinase. Diabetes.

[13] Scheele C, Nielsen S, Kelly M, Broholm C, Nielsen AR, Taudorf S, Pedersen M, Fischer CP, Pedersen BK (2012). Satellite cells derived from obese humans with type 2 diabetes and differentiated into myocytes in vitro exhibit abnormal response to IL-6. PloS One.

[14] Broholm C, Brandt C, Schultz NS, Nielsen AR, Pedersen BK, Scheele C (2012). Deficient leukemia inhibitory factor signaling in muscle precursor cells from patients with type 2 diabetes. Am J Physiol Endocrinol Metab.

[15] Ling C, Poulsen P, Simonsson S, Ronn T, Holmkvist J, Almgren P, Hagert P, Nilsson E, Mabey AG, Nilsson P (2007). Genetic and epigenetic factors are associated with expression of respiratory chain component NDUFB6 in human skeletal muscle. J Clin Investig.

[16] Ronn T, Poulsen P, Hansson O, Holmkvist J, Almgren P, Nilsson P, Tuomi T, Isomaa B, Groop L, Vaag A (2008). Age influences DNA methylation and gene expression of COX7A1 in human skeletal muscle. Diabetologia.

[17] Nitert MD, Dayeh T, Volkov P, Elgzyri T, Hall E, Nilsson E, Yang BT, Lang S, Parikh H, Wessman Y (2012). Impact of an exercise intervention on DNA methylation in skeletal muscle from first-degree relatives of patients with type 2 diabetes. Diabetes.

[18] Jacobsen SC, Brons C, Bork-Jensen J, Ribel-Madsen R, Yang B, Lara E, Hall E, Calvanese V, Nilsson E, Jorgensen SW (2012). Effects of short-term high-fat overfeeding on genome-wide DNA methylation in the skeletal muscle of healthy young men. Diabetologia.

[19] Jacobsen SC, Gillberg L, Bork-Jensen J, Ribel-Madsen R, Lara E, Calvanese V, Ling C, Fernandez AF, Fraga MF, Poulsen P (2014). Young men with low birthweight exhibit decreased plasticity of genome-wide muscle DNA methylation by high-fat overfeeding. Diabetologia.

[20] Ribel-Madsen R, Fraga MF, Jacobsen S, Bork-Jensen J, Lara E, Calvanese V, Fernandez AF, Friedrichsen M, Vind BF, Hojlund K (2012). Genome-wide analysis of DNA methylation differences in muscle and fat from monozygotic twins discordant for type 2 diabetes. PloS One.

[21] Castiglioni A, Hettmer S, Lynes MD, Rao TN, Tchessalova D, Sinha I, Lee BT, Tseng YH, Wagers AJ (2014). Isolation of progenitors that exhibit myogenic/osteogenic bipotency in vitro by fluorescence-activated cell sorting from human fetal muscle. Stem Cell Rep.

[22] Bibikova M, Barnes B, Tsan C, Ho V, Klotzle B, Le JM, Delano D, Zhang L, Schroth GP, Gunderson KL (2011). High density DNA methylation array with single CpG site resolution. Genomics.

[23] Wang J, Duncan D, Shi Z, Zhang B (2013). WEB-based GEne SeT AnaLysis Toolkit (WebGestalt): update 2013. Nucleic Acids Res.

[24] Hall E, Volkov P, Dayeh T, Esguerra JL, Salo S, Eliasson L, Ronn T, Bacos K, Ling C (2014). Sex differences in the genome-wide DNA methylation pattern and impact on gene expression, microRNA levels and insulin secretion in human pancreatic islets. Genome Biol.

[25] Dmitriev P, Barat A, Polesskaya A, O'Connell MJ, Robert T, Dessen P, Walsh TA, Lazar V, Turki A, Carnac G (2013). Simultaneous miRNA and mRNA transcriptome profiling of human myoblasts reveals a novel set of myogenic differentiation-associated miRNAs and their target genes. BMC Genomics..

[26] Lister R, Pelizzola M, Dowen RH, Hawkins RD, Hon G, Tonti-Filippini J, Nery JR, Lee L, Ye Z, Ngo QM (2009). Human DNA methylomes at base resolution show widespread epigenomic differences. Nature.

[27] Subramanian A, Tamayo P, Mootha VK, Mukherjee S, Ebert BL, Gillette MA, Paulovich A, Pomeroy SL, Golub TR, Lander ES (2005). Gene set enrichment analysis: a knowledge-based approach for interpreting genome-wide expression profiles. Proc Natl Acad Sci U S A.

[28] Pedersen BK, Febbraio MA (2012). Muscles, exercise and obesity: skeletal muscle as a secretory organ. Nat Rev Endocrinol.

[29] Martinez FF, Cervi L, Knubel CP, Panzetta-Dutari GM, Motran CC (2013). The role of pregnancy-specific glycoprotein 1a (PSG1a) in regulating the innate and adaptive immune response. Am J Reprod Immunol.

[30] Fournier T, Guibourdenche J, Evain-Brion D (2015). hCGs: Different sources of production, different glycoforms and functions. Placenta..

[31] Zambelli F, Pesole G, Pavesi G (2009). Pscan: finding over-represented transcription factor binding site motifs in sequences from co-regulated or co-expressed genes. Nucleic Acids Res.

[32] Mathelier A, Zhao X, Zhang AW, Parcy F, Worsley-Hunt R, Arenillas DJ, Buchman S, Chen CY, Chou A, Ienasescu H (2014). JASPAR 2014: an extensively expanded and updated open-access database of transcription factor binding profiles. Nucleic Acids Res.

[33] Jones PA (2012). Functions of DNA methylation: islands, start sites, gene bodies and beyond. Nat Rev Genet.

[34] Joosten LA, Heinhuis B, Netea MG, Dinarello CA (2013). Novel insights into the biology of interleukin-32. Cell Mol Life Sci.

[35] Choi J, Bae S, Hong J, Ryoo S, Jhun H, Hong K, Yoon D, Lee S, Her E, Choi W (2010). Paradoxical effects of constitutive human IL-32{gamma} in transgenic mice during experimental colitis. Proc Natl Acad Sci U S A.

[36] Getz GS, Reardon CA (2006). Diet and murine atherosclerosis. Arterioscler Thromb Vasc Biol.

[37] Akhmedov D, Berdeaux R (2013). The effects of obesity on skeletal muscle regeneration. Front Physiol..

[38] Warde-Farley D, Donaldson SL, Comes O, Zuberi K, Badrawi R, Chao P, Franz M, Grouios C, Kazi F, Lopes CT (2010). The GeneMANIA prediction server: biological network integration for gene prioritization and predicting gene function. Nucleic Acids Res.

[39] Shannon P, Markiel A, Ozier O, Baliga NS, Wang JT, Ramage D, Amin N, Schwikowski B, Ideker T (2003). Cytoscape: a software environment for integrated models of biomolecular interaction networks. Genome Res.

[40] Dayeh TA, Olsson AH, Volkov P, Almgren P, Rönn T, Ling C (2013). Identification of CpG-SNPs associated with type 2 diabetes and differential DNA methylation in human pancreatic islets. Diabetologia.

[41] Henningsen J, Rigbolt KT, Blagoev B, Pedersen BK, Kratchmarova I (2010). Dynamics of the skeletal muscle secretome during myoblast differentiation. Mol Cell Proteomics.

[42] Ryall JG, Dell'Orso S, Derfoul A, Juan A, Zare H, Feng X, Clermont D, Koulnis M, Gutierrez-Cruz G, Fulco M (2015). The NAD(+)-dependent SIRT1 deacetylase translates a metabolic switch into regulatory epigenetics in skeletal muscle stem cells. Cell Stem Cell.

[43] Austin L, Burgess AW (1991). Stimulation of myoblast proliferation in culture by leukaemia inhibitory factor and other cytokines. J Neurol Sci.

[44] White JD, Bower JJ, Kurek JB, Austin L (2001). Leukemia inhibitory factor enhances regeneration in skeletal muscles after myoblast transplantation. Muscle Nerve.

[45] Broholm C, Laye MJ, Brandt C, Vadalasetty R, Pilegaard H, Pedersen BK, Scheele C (2011). LIF is a contraction-induced myokine stimulating human myocyte proliferation. J Appl Physiol.

[46] Barker T, Traber MG (2007). From animals to humans: evidence linking oxidative stress as a causative factor in muscle atrophy. J Physiol.

[47] Apostolova MD, Ivanova IA, Cherian MG (2000). Signal transduction pathways, and nuclear translocation of zinc and metallothionein during differentiation of myoblasts. Biochem Cell Biol.

[48] Russell AP, Hesselink MK, Lo SK, Schrauwen P (2005). Regulation of metabolic transcriptional co-activators and transcription factors with acute exercise. FASEB J.

[49] Engeli S, Birkenfeld AL, Badin PM, Bourlier V, Louche K, Viguerie N, Thalamas C, Montastier E, Larrouy D, Harant I (2012). Natriuretic peptides enhance the oxidative capacity of human skeletal muscle. J Clin Investig.

[50] Soeters MR, Soeters PB (2012). The evolutionary benefit of insulin resistance. Clin Nutr.

[51] Rodriguez J, Vernus B, Chelh I, Cassar-Malek I, Gabillard JC, Hadj Sassi A, Seiliez I, Picard B, Bonnieu A (2014). Myostatin and the skeletal muscle atrophy and hypertrophy signaling pathways. Cell Mol Life Sci.

[52] Artaza JN, Bhasin S, Mallidis C, Taylor W, Ma K, Gonzalez-Cadavid NF (2002). Endogenous expression and localization of myostatin and its relation to myosin heavy chain distribution in C2C12 skeletal muscle cells. J Cell Physiol.

[53] Allen DL, Hittel DS, McPherron AC (2011). Expression and function of myostatin in obesity, diabetes, and exercise adaptation. Med Sci Sports Exerc.

[54] Camporez JG, Petersen MC, Abudukadier A, Moreira GV, Jurczak MJ, Friedman G, Haqq CM, Petersen KF, Shulman GI (2016). Anti-myostatin antibody increases muscle mass and strength and improves insulin sensitivity in old mice. Proc Natl Acad Sci U S A.

[55] Baylin SB, Jones PA (2011). A decade of exploring the cancer epigenome - biological and translational implications. Nat Rev Cancer.

[56] McCarthy MI (2010). Genomics, type 2 diabetes, and obesity. N Engl J Med.

[57] Rönn T, Volkov P, Gillberg L, Kokosar M, Perfilyev A, Jacobsen AL, Jorgensen SW, Brons C, Jansson PA, Eriksson KF (2015). Impact of age, BMI and HbA1c levels on the genome-wide DNA methylation and mRNA expression patterns in human adipose tissue and identification of epigenetic biomarkers in blood. Hum Mol Genet.

[58] Ronn T, Volkov P, Davegardh C, Dayeh T, Hall E, Olsson AH, Nilsson E, Tornberg A, Dekker Nitert M, Eriksson KF (2013). A six months exercise intervention influences the genome-wide DNA methylation pattern in human adipose tissue. PLoS Genet.

[59] Olsson AH, Volkov P, Bacos K, Dayeh T, Hall E, Nilsson EA, Ladenvall C, Ronn T, Ling C (2014). Genome-wide associations between genetic and epigenetic variation influence mRNA expression and insulin secretion in human pancreatic islets. PLoS Genet.

[60] Nilsson E, Jansson PA, Perfilyev A, Volkov P, Pedersen M, Svensson MK, Poulsen P, Ribel-Madsen R, Pedersen NL, Almgren P (2014). Altered DNA methylation and differential expression of genes influencing metabolism and inflammation in adipose tissue from subjects with type 2 diabetes. Diabetes.

[61] Dayeh T, Volkov P, Salo S, Hall E, Nilsson E, Olsson AH, Kirkpatrick CL, Wollheim CB, Eliasson L, Ronn T (2014). Genome-wide DNA methylation analysis of human pancreatic islets from type 2 diabetic and non-diabetic donors identifies candidate genes that influence insulin secretion. PLoS Genet.

[62] Jeltsch A, Jurkowska RZ (2014). New concepts in DNA methylation. Trends Biochem Sci.

[63] Trowbridge JJ, Snow JW, Kim J, Orkin SH (2009). DNA methyltransferase 1 is essential for and uniquely regulates hematopoietic stem and progenitor cells. Cell Stem Cell.

[64] Robinson MR, Wray NR, Visscher PM (2014). Explaining additional genetic variation in complex traits. Trends Genet.

[65] Faenza I, Bavelloni A, Fiume R, Lattanzi G, Maraldi NM, Gilmour RS, Martelli AM, Suh PG, Billi AM, Cocco L (2003). Up-regulation of nuclear PLCbeta1 in myogenic differentiation. J Cell Physiol.

[66] Giacinti C, Musaro A, De Falco G, Jourdan I, Molinaro M, Bagella L, Simone C, Giordano A (2008). Cdk9-55: a new player in muscle regeneration. J Cell Physiol.

[67] Bruun JM, Stallknecht B, Helge JW, Richelsen B (2007). Interleukin-18 in plasma and adipose tissue: effects of obesity, insulin resistance, and weight loss. Eur J Endocrinol.

[68] Badin PM, Louche K, Mairal A, Liebisch G, Schmitz G, Rustan AC, Smith SR, Langin D, Moro C (2011). Altered skeletal muscle lipase expression and activity contribute to insulin resistance in humans. Diabetes.

[69] Butler AA, Tam CS, Stanhope KL, Wolfe BM, Ali MR, O'Keeffe M, St-Onge MP, Ravussin E, Havel PJ (2012). Low circulating adropin concentrations with obesity and aging correlate with risk factors for metabolic disease and increase after gastric bypass surgery in humans. J Clin Endocrinol Metab.

[70] Pihlajamaki J, Boes T, Kim EY, Dearie F, Kim BW, Schroeder J, Mun E, Nasser I, Park PJ, Bianco AC (2009). Thyroid hormone-related regulation of gene expression in human fatty liver. J Clin Endocrinol Metab.

[71] Kobayashi M, Bai YP, Dong YS, Yu H, Chen SS, Gao R, Zhang LJ, Yoder MC, Kapur R, Zhang ZY (2014). PRL2/PTP4A2 phosphatase is important for hematopoietic stem cell self-renewal. Stem Cells.

[72] Will CL, Schneider C, Hossbach M, Urlaub H, Rauhut R, Elbashir S, Tuschl T, Luhrmann R (2004). The human 18S U11/U12 snRNP contains a set of novel proteins not found in the U2-dependent spliceosome. RNA.

