# Supplementary material for: Abnormal epigenetic changes during differentiation of human skeletal muscle stem cells from obese subjects
Source: BMC Med. 2017 Feb 22;15:39. doi: 10.1186/s12916-017-0792-x (PMC5320752; doi:10.1186/s12916-017-0792-x)
Supplement: Additional file 1: — Supplemental methods. Table S12. Antibodies used for western blot. Figure S1. Characterization of the primary human myoblasts. Figure S2. Silencing of ARPP21, SMAD6, and PLAC8 during differentiation of primary human myoblasts. Figure S3. Differences in methylation, transcription, and secreted cytokine levels before versus after differentiation of primary human myoblast from obese and non-obese subjects. (PDF 1126 kb) [file 12916_2017_792_MOESM1_ESM.pdf]

SUPPLEMENTAL INFORMATION

SUPPLEMANTAL METHODS

Study participants

We utilized material from a sub-set of a previously described human cohort [1, 2]. The participants were divided into distinct groups according to BMI, categorized as non-obese or obese (**Table 1**).

Height and weight were measured using standard techniques. Measures of body composition were estimated using dual-energy x-ray absorptiometry (Lunar iDXA, GE Healthcare, Madison, WI). A standardized OGTT was performed. Physical fitness was assessed by a single-stage sub-maximal model (Aastrand test) [3]. All study participants had provided informed written consent and the study was approved by the local ethics committees and conducted in accordance with the principles of the Helsinki Declaration.

Primary human muscle stem cell isolation and culture of myoblasts

Primary muscle stem cells (satellite cells) were isolated from human skeletal muscle biopsies obtained under local anesthesia from the vastus lateralis muscle using a biopsy needle with suction as described in detail previously [4].

Unless otherwise stated, all cell culture reagents were from Invitrogen, Carlsbad, CA, USA. Isolated satellite cells were cultured in growth media (HAM/F10 supplied with 20% Fetal Bovine Serum (FBS) and 1% penicillin/streptomycin). Myoblasts were harvested when less than 50% confluent (**Figure S1A**). For differentiation of myoblasts into myotubes, the myoblasts were grown until 70-80% confluency and then the media was changed to differentiation media 1 (DMEM 1.0 g/L glucose supplied with 10% FBS and 1% penicillin/streptomycin) for two-three days, until the myoblasts were completely confluent. Hereafter, media was changed to differentiation media 2 (DMEM 4.5 g/L glucose supplied with 2% horse serum and 1% penicillin/streptomycin) thereby initiating fusion into myotubes (**Figure S1A**). Cultures

were fully differentiated by day 5 as determined by visual confirmation of myotube formation (>3 nuclei per myotube in ~70% of the cells) (**Figure S1A**). Two hours before harvesting of RNA the media was changed to DMEM 1.0 g/L glucose without any supplements.

RNA and DNA extraction for arrays

RNA was extracted from cells by Trizol (Invitrogen) followed by RNeasy MinElute Cleanup kit (Qiagen). Genomic DNA was extracted from cells using the DNeasy blood and tissue kit (Qiagen). Nucleic acid quantity and purity were determined using a NanoDrop 1000 spectrophotometer (NanoDrop Technologies, Wilmington, DE, USA) and RNA integrity using the Bioanalyzer system (Agilent, Santa Clara, CA, USA).

DNA methylation arrays

DNA methylation was analyzed using Infinium HumanMethylation450 BeadChip (Illumina, San Diego, CA, USA). This array contains 485,577 probes, which cover 21,231 (99%) RefSeq genes [5]. DNA was first bisulfite treated using the EZ DNA methylation kit (Zymo Research, Orange, CA, USA). Analysis of DNA methylation with Infinium®assay was carried out on bisulfite-converted DNA, with all procedures following the standard Infinium HD Assay Methylation Protocol Guide (Illumina #15019519). To minimize batch effects, paired samples, i.e. myoblasts and myotubes from one subject, were always hybridized on the same chip and non-obese and obese subjects were evenly distributed on the chips. Bioinformatics analyses were performed as previously described [6], but without omitting quintile normalization in calculations of global methylation. Additionally, probes with a mean detection p-value >0.01, probes covering the Y-chromosome and non-CpG sites as well as rs-probes detecting SNPs were filtered away from the main analysis. The raw methylation score for each analyzed site is presented as β-value ranging from 0 to 1 (0-100% methylation).

Non-CpG sites were analyzed separately. The Infinium HumanMethylation450 BeadChip covers 3,091 non-CpG sites, but many of these are analyzed by probes that may cross-react to other parts in the genome [7]. After filtering out cross-reactive probes and QC, methylation data for 1,101 non-CpG sites remained.

mRNA expression arrays

mRNA expression was analyzed using HumanHT-12 Expression BeadChip (Illumina) according to the manufacturer's recommendations. This array contains 47,231 probes covering 28,688 well-annotated coding transcripts. Probes with mean detection p-value>0.01 for more than 60% of the samples were filtered out and data were background corrected, log2 transformed and quintile normalized. Batch correction using COMBAT [8] was performed for non-paired statistical analyses. Expression levels in tables and figures are presented as un-logarithmic values. One sample of non-obese and one sample of obese did not pass the QC and were removed from the analyses, wherefore expression data of 13 subjects in each group are presented.

Flow cytometry for determination of muscle precursor cell purity

Myogenic purity of the primary human skeletal muscle cell culture was analyzed by flow cytometry. The expression of the cell surface markers CD56, CD31, and CD45 were measured. Isolated cells were propagated in growth media until 70-80% confluence. Myoblasts were detached using TrypLE and then washed twice in washing buffer (PBS containing 2% FBS and 0.01% NaN_3) and once in staining buffer (PBS containing 2% FBS, 1% human serum, and 0.01% NaN3). Myoblasts were stained with anti-human CD56-APC, CD31-PE, and CD45-BV421 (all from BD Bioscience, Franklin Lakes, NJ, USA) for 20 minutes and subsequently washed three times in wash buffer. Data were acquired using a FACSFortessa (BD Biosciences). For compensation, single stain was used with one drop of negative control beads and anti-mouse IgG beads (BD Biosciences). Data analysis was performed using Kaluza software version 1.2 (Beckman Coulter, Brea, CA, USA).

Pathway analyses

Webgestalt [9] was used to analyze enriched KEGG pathways for genes with one or more differentially methylated CpG site(s), while expression data, where genes can be ranked based on expression direction, were analyzed with GSEA [10]. Webgestalt was also used to search for enriched GO-terms for CpG sites only differentially methylated in obese. Accession numbers for all significant CpG sites ($q<0.05$) were used for gene identification in WebGestalt. Accession numbers for all analyzed CpG sites were used as reference. Benjamini–Hochberg correction was used to adjust for multiple testing and the minimum number of genes per pathway required was two. For GSEA was the expression of all analyzed transcripts on the array ranked according to t-statistics in a paired t-test comparing myoblasts with myotubes. The analysis was run with highest occurrence for genes with multiple probes. Pathways with 1-500 transcripts were considered.

PSCAN

PSCAN Web Interface [11] together with JASPAR [12] were used to find enriched transcription binding motifs 0-1,000 bp upstream of transcription start sites of differentially expressed genes. Bonferroni-corrected significance threshold was used.

GeneMANIA Cytoscape Plugin

Gene symbols were imported to Cytoscape with GeneMANIA Cytoscape plugin [13, 14]. A network was created based on the category called pathway networks. Genes contributing to processes presented in the figures were selected (**Table S8**) and new networks created.

Luciferase assay

A 2000 bp promoter fragment upstream of the *MCM10* transcription start site was inserted into a CpG-free luciferase reporter vector (pCpGL-basic) and *in vitro* methylated with SssI (New England Biolabs, Frankfurt, Germany) as described elsewhere [6]. HeLa cells were transfected with 100 ng of methylated or mock-methylated construct together with 4 ng pRL renilla luciferase control reporter vector as a control

for transfection efficiency (pRL-CMV vector, Promega, Madison, WI, USA). Firefly and renilla luciferase luminescence were measured with Dual-Glo® Luciferase Assay System (Promega) and an Infinite® M200 PRO multiplate reader (Tecan Group Ltd, Männedorf, Switzerland). The results represent the mean of four independent experiments analyzed in triplicate. Cells transfected with an empty pCpGL-vector were used as background control in each experiment.

Transfection with siRNA

Primary myoblasts were transfected with ON-TARGET plus human siRNA SMART pool (Dharmacon, Lafayette, CO, USA) targeting *IL32* (J-015988-05/06/07/08), *ARPP21* (J-016091-17/18/19/20), *SMAD6* (J-015362-05/06/07/08*), PLAC8* (J-020311-09/10/11/12), *DNMT1* (J-004605-06/07/08/09) or a negative control (Non-targeting plus #D-001810-10-05). siRNA, corresponding to a final concentration of 50 nM, was mixed with Opti-Mem reduced serum media (Gibco, cat #31985-062) and Lipofectamine RNAiMAX (Invitrogen, #13778-075) (7.5 µl/well) and incubated for 20 minutes in RT. 0.5 ml siRNA/Lipofectamine was added to cells in 2 ml penicillin-free cell culture media. *IL32, ARPP21* and *DNMT1* were silenced at differentiation start and harvested after 3 and 7 days (**Figure S2B**). *SMAD6* and *PLAC8* were silenced one day before induction of differentiation, in 75-85% confluent cells and harvested after 2 and 8 days (**Figure S2J**). Cells transfected with control siRNA were harvested at the same time point as the cells subjected to gene silencing. The siRNA experiments were performed in human myoblasts from the Centre of Inflammation and Metabolism and the Centre for Physical Activity Research (University of Copenhagen, Denmark) or Cook Myosite (Pittsburgh, PA, USA).

qPCR

RNA was extracted using miRNeasy MiniKit (Qiagen) and converted to cDNA with QuantiTect Reverse Transcriptase Kit (Qiagen). qPCR was run with pre-designed TaqMan Gene Expression assays (Applied Biosystems) for *ARPP21* (Hs01020723_m1), *IL32* (Hs00992441_m1), *SMAD6* (Hs00178579_m1), *PLAC8* (Hs00930964_g1), *DNMT1* (Hs00945875_m1), *DNMT3A* (Hs01027166_m1) and *DNMT3B*

(Hs00171876_m1) or SYBRgreen primers (DNA Technology A/S Risskov, Denmark) for *cMYC* (f5'GATCCAGACTCTGACCTTTTGC, r5' CACCAGCAGCGACTCTGA), *JUNB* (f5'GCTCGGTTTCAGGAGTTTGT, R5'ATACACAGCTACGGGATACGG, *MYOD1* (f5'CACTACAGCGGGCGACTCC, r5'TAGGCGCCTTCGTAGCAG), *MYOG* (f5'GCTCAGCTCCCTCAACCA, r5'GCTGTGAGAGCTGCATTCG) and *TNNI1* (f5'GGCCAACCTCAAGTCTGTG, r5'AGACATGGCCTCCACGTT) and detected with ViiATM7 Real-Time PCR system (Applied Biosystems). Samples were run in triplicate and quantified using the standard curve method. Expression levels were normalized to *PPIA* (Applied Biosystems #4326316E-0901011).

AKT phosphorylation and Western Blot analysis

siRNA transfected cells were incubated with or without 100 nM insulin for 30 minutes on day 7 of differentiation. Cells were lysed in ice-cold cell lysis buffer (20 mM TRIS-HCl, 1 mM EDTA, 1 mM EGTA, 1% Triton X-100, 150 mM NaCl, and 1 mM Na_3VO_4) with protease inhibitor cocktail, phosphatase inhibitor 2 and phosphatase inhibitor 3. Lysates were centrifuged at 10,000g for 5 minutes and protein concentration of supernatants determined with a Bicinchoninic acid (BCA) assay. 10 µg of protein were mixed with sample buffer, loaded on to electrophoresis gels and transferred to PVDF membranes (0.2 µm). After blocking, the membranes were incubated with a primary antibody overnight (**Table S12**), followed by secondary antibody for 1 hour in RT (**Table S12**). IL-32 and DNMT1 protein levels were normalized to total protein using stain-free technology (Bio-Rad V3 Western Workflow) and pAKT were normalized to total AKT.

ATP assay

Differentiated myotubes were serum starved for 2 hours in DMEM 1.0 g/l glucose, incubated in KRH media (140 mM NaCl, 20 mM HEPES, 5 mM KCl, 2.5 mM $MgSO_4*7H_2O$, 1 mM $CaCl_2*2H_2O$, pH 7.5) for 30 minutes and then lysed in ice cold RIPA buffer (50 mM Tris-HCl, 150 mM NaCl, 2 mM EDTA, 1% TritonX, 0.5% Na-deoxycholate, 0.1% SDS) with a protease inhibitor cocktail. All cell lysates where

frozen before analyzing ATP levels in triplicates with ATP kit SL (144-041 Biothema, Handen, Sweden) according to the manufacturer's instructions.

IL32 expression data from human muscle biopsies

IL32 mRNA expression in human muscle biopsies was analyzed by microarray as previously described [15]. This cohort includes middle aged, sedentary and healthy men. Muscle biopsies were taken from vastus lateralis in the fasted state. HOMA-IR was analyzed based on their fasting glucose and insulin levels.

IL32tg mice

*IL32*tg mice were generated as previously described [16] (n=9) and WT C57BL/6 mice were used as control (n=10). At the age of 16 weeks the mice were fed a high fat diet known as paigen diet [17] for 18 weeks when animals were sacrificed and tibialis anterior and soleus excised. The OGTT and ITT were performed after 16 and 17 weeks of HFD, respectively. Here, 2 g/kg glucose or 0.75 U/kg insulin were administrated in the fasting state, respectively. Blood samples were taken before and at 20, 40, 60, 90 and 120 minutes for the OGTT and before and at 20 minutes for the ITT. Glucose levels were measured with a glucometer (Accu-Chek, Roche Diagnostics).

For *in vitro* incubations, fresh soleus muscle from 5 mice in each group (*IL32*tg and WT) were pre-incubated for 10 minutes in Krebs Ringer Buffer (KRB) (117 mM NaCl, 4.7 mM KCl, 2.5 mM CaCl2, 1.2 mM KH2PO4, 1.2 mM MgSO4, 0.5 mM NaHCO3 (pH 7.4)) supplemented with 0.1% Bovine Serum Albumin (BSA), 8 mM mannose and 2 mM pyruvate. Thereafter, the muscles were incubated in KRB in the absence or presence of a submaximal insulin concentration (50 μU/ml) for 30 minutes and frozen in liquid nitrogen. Muscle processing was then performed as previously described [18] followed by Western blot analysis (see above). One WT muscle sample was excluded since the *in vitro* incubation failed and the insulin response was reversed according to Akt phosphorylation data.

Gene expression was analyzed in tibialis from 6 mice in each group (*IL32*tg and WT) using MouseWG-6 v2.0 Expression BeadChip array (Illumina) according to the manufacturer's recommendations. Probes with mean detection p-value>0.01 for more than 80% of the samples were filtered out and data were background corrected, log2 transformed and quintile normalized.

All animal experiments were approved by the National Jewish Health (NJH) and Institutional Animal Care and Use Committee (IACUC).

Cytokine secretion analysis

Medium collected during cell culture were analyzed in duplicate with ELISA to detect CCL2, TNF-α, IL-6 (K151AYB-1, K151BHB-1 and K151AKB-1, MesoScale Discovery, Rockville, MD, USA), TGF-β3 (#MBS2021763, MyBioSource) and IL-32 (#DY3040-05, R&D Systems) according to the manufactures' instructions.

SUPPLEMENTAL REFERENCES

1. Green CJ, Pedersen M, Pedersen BK, Scheele C: **Elevated NF-kappaB activation is conserved in human myocytes cultured from obese type 2 diabetic patients and attenuated by AMP-activated protein kinase**. *Diabetes* 2011, **60**(11):2810-2819.

2. Varemo L, Scheele C, Broholm C, Mardinoglu A, Kampf C, Asplund A, Nookaew I, Uhlen M, Pedersen BK, Nielsen J: **Proteome- and transcriptome-driven reconstruction of the human myocyte metabolic network and its use for identification of markers for diabetes**. *Cell reports* 2015, **11**(6):921-933.

3. Astrand PO, Ryhming I: **A nomogram for calculation of aerobic capacity (physical fitness) from pulse rate during sub-maximal work**. *J Appl Physiol* 1954, **7**(2):218-221.

4. Broholm C, Brandt C, Schultz NS, Nielsen AR, Pedersen BK, Scheele C: **Deficient leukemia inhibitory factor signaling in muscle precursor cells from patients with type 2 diabetes**. *American journal of physiology Endocrinology and metabolism* 2012, **303**(2):E283-292.

5. Bibikova M, Barnes B, Tsan C, Ho V, Klotzle B, Le JM, Delano D, Zhang L, Schroth GP, Gunderson KL *et al*: **High density DNA methylation array with single CpG site resolution**. *Genomics* 2011, **98**(4):288-295.

6. Ronn T, Volkov P, Davegardh C, Dayeh T, Hall E, Olsson AH, Nilsson E, Tornberg A, Dekker Nitert M, Eriksson KF *et al*: **A six months exercise intervention influences the genome-wide DNA methylation pattern in human adipose tissue**. *PLoS genetics* 2013, **9**(6):e1003572.

7. Chen YA, Lemire M, Choufani S, Butcher DT, Grafodatskaya D, Zanke BW, Gallinger S, Hudson TJ, Weksberg R: **Discovery of cross-reactive probes and polymorphic CpGs in the Illumina Infinium HumanMethylation450 microarray**. *Epigenetics : official journal of the DNA Methylation Society* 2013, **8**(2):203-209.

8. Johnson WE, Li C, Rabinovic A: **Adjusting batch effects in microarray expression data using empirical Bayes methods**. *Biostatistics* 2007, **8**(1):118-127.

9. Wang J, Duncan D, Shi Z, Zhang B: **WEB-based GEne SeT AnaLysis Toolkit (WebGestalt): update 2013**. *Nucleic acids research* 2013, **41**(Web Server issue):W77-83.

10. Subramanian A, Tamayo P, Mootha VK, Mukherjee S, Ebert BL, Gillette MA, Paulovich A, Pomeroy SL, Golub TR, Lander ES *et al*: **Gene set enrichment analysis: a knowledge-based approach for interpreting genome-wide expression profiles**. *Proceedings of the National Academy of Sciences of the United States of America* 2005, **102**(43):15545-15550.

11. Zambelli F, Pesole G, Pavesi G: **Pscan: finding over-represented transcription factor binding site motifs in sequences from co-regulated or co-expressed genes**. *Nucleic acids research* 2009, **37**(Web Server issue):W247-252.

12. Mathelier A, Zhao X, Zhang AW, Parcy F, Worsley-Hunt R, Arenillas DJ, Buchman S, Chen CY, Chou A, Ienasescu H *et al*: **JASPAR 2014: an extensively expanded and updated open-access database of transcription factor binding profiles**. *Nucleic acids research* 2014, **42**(Database issue):D142-147.

13. Warde-Farley D, Donaldson SL, Comes O, Zuberi K, Badrawi R, Chao P, Franz M, Grouios C, Kazi F, Lopes CT *et al*: **The GeneMANIA prediction server: biological network integration for gene prioritization and predicting gene function**. *Nucleic acids research* 2010, **38**(Web Server issue):W214-220.

14. Shannon P, Markiel A, Ozier O, Baliga NS, Wang JT, Ramage D, Amin N, Schwikowski B, Ideker T: **Cytoscape: a software environment for integrated models of biomolecular interaction networks**. *Genome research* 2003, **13**(11):2498-2504.

15. Nitert MD, Dayeh T, Volkov P, Elgzyri T, Hall E, Nilsson E, Yang BT, Lang S, Parikh H, Wessman Y *et al*: **Impact of an exercise intervention on DNA methylation in skeletal muscle from first-degree relatives of patients with type 2 diabetes**. *Diabetes* 2012, **61**(12):3322-3332.

16. Choi J, Bae S, Hong J, Ryoo S, Jhun H, Hong K, Yoon D, Lee S, Her E, Choi W *et al*: **Paradoxical effects of constitutive human IL-32{gamma} in transgenic mice during experimental colitis**. *Proceedings of the National Academy of Sciences of the United States of America* 2010, **107**(49):21082-21086.

17. Getz GS, Reardon CA: **Diet and murine atherosclerosis**. *Arterioscler Thromb Vasc Biol* 2006, **26**(2):242-249.

18. Fentz J, Kjobsted R, Birk JB, Jordy AB, Jeppesen J, Thorsen K, Schjerling P, Kiens B, Jessen N, Viollet B *et al*: **AMPK alpha is critical for enhancing skeletal muscle fatty acid utilization during in vivo exercise in mice**. *Faseb Journal* 2015, **29**(5):1725-1738.

SUPPLEMENTAL TABLES

Table S12. Antibodies used for western blot.

Target	Antibody	Dilution	Blocking	Secondary antibody
P-AKT (Ser473)	#9271, Cell signaling	1:1000 in 1% fish skin gelatin (FSG)/TBST	2.5% milk/TBST	#P0448, Dako 1:5000 in 2.5% milk/TBST
P-AKT (Thr308)	#9275, Cell signaling	1:5,000 in 5% BSA/TBST	2% milk/TBST	#111-035-045, Jackson ImmunoResearch 1:5000 in 2% BSA/TBST
AKT2	#3063, Cell signaling	1:1000 in 5% BSA/TBST	2% milk/TBST	#111-035-045, Jackson IR 1:5000 in 2% BSA/TBST
Total-AKT	#9272, Cell signaling	1:2000 in 5% BSA/TBST	5% milk/TBST	#7074, Cell signaling 1:10000 in 5% milk/TBST
DNMT1	#NB100-264SS, Novus Biologicals	1:1000 in 5% BSA/TBST	5% BSA/TBST	#7074, Cell signaling 1:10000 in 5% milk/TBST
IL-32	#AF3040, R&D Systems	0.2 µg/ml in TBST	5% milk/TBST	#HAF109 R&D, Systems 1:5000 in 5% milk/TBST

SUPPLEMENTAL FIGURES

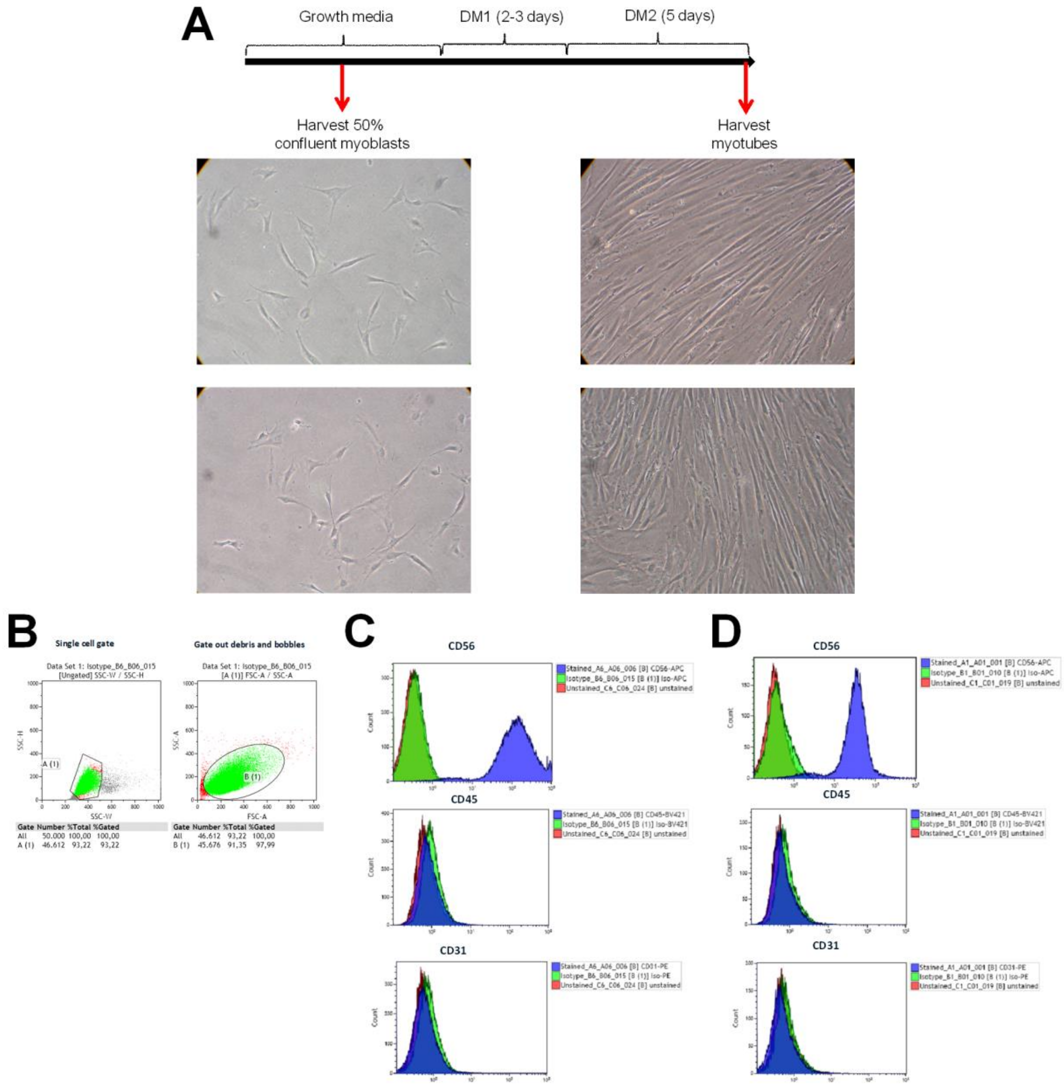

Figure S1. Characterization of the primary human myoblasts

A) Experimental set-up showing the differentiation of myoblasts, together with pictures of both myoblasts and myotubes.

B-D) Cells isolated from muscle biopsies obtained from obese and non-obese individuals were cultured and propagated until 70-80% confluence to determine purity of the myoblast cultures by flow cytometry. Single cells were gated in a SSC-H vs SSC-W scatter (gate a), followed by a SSC vs FSC scatter where

debris was gated out (gate b) (B). For the cells analyzed in gate b, representative histograms are shown for myoblasts isolated from non-obese individuals (C) and myoblasts isolated from obese individuals (D). Red histograms represent un-stained cells and green histograms represent cells stained with the corresponding isotype. Blue histograms represent cells stained with the indicated markers. SSC-H, Side scatter pulse height; SSC-W, Side scatter pulse width; FSC, Forward scatter.

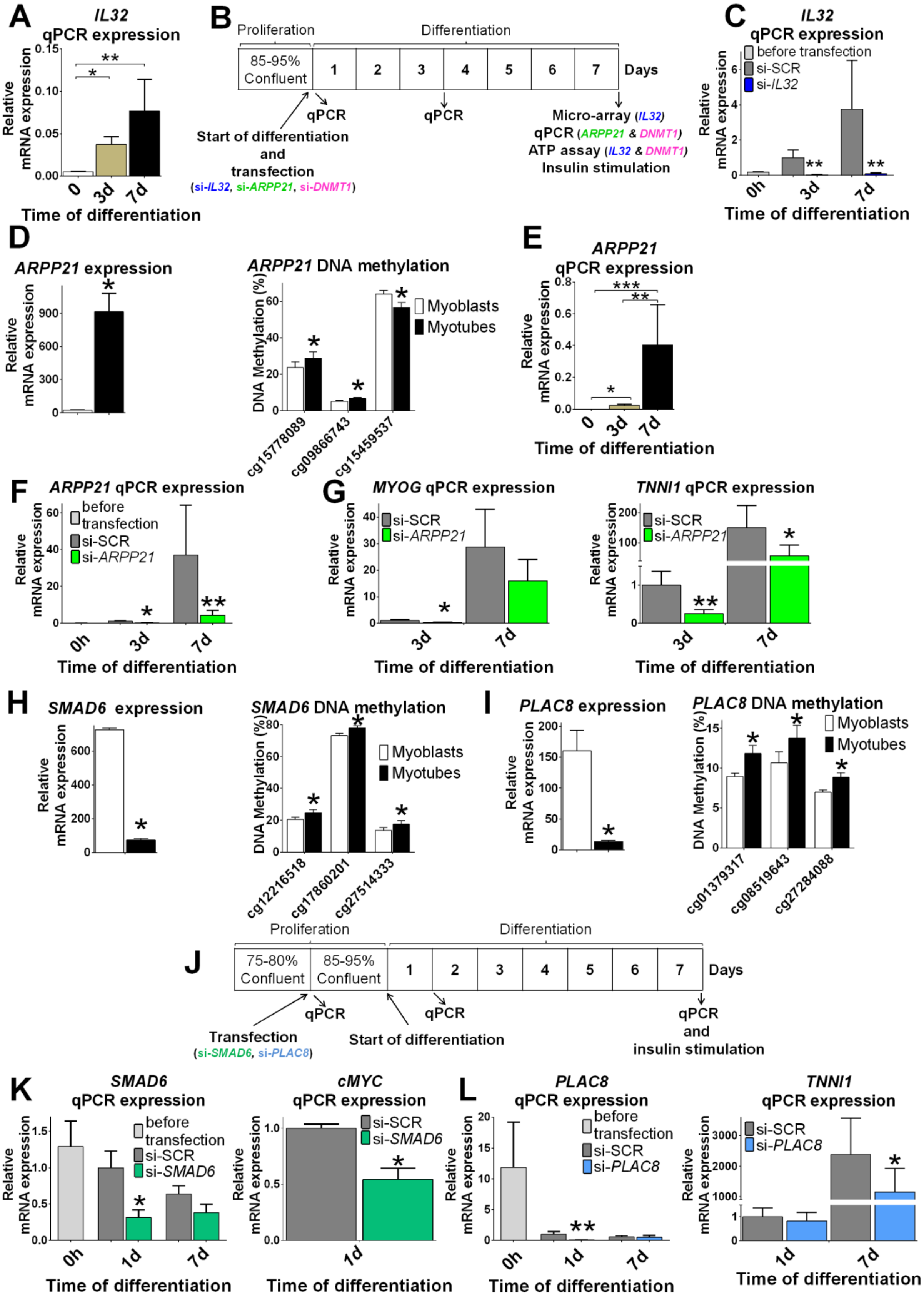
A
IL32 qPCR expression
Relative mRNA expression
Time of differentiation
B
Proliferation
Differentiation
85-95% Confluent
Days
qPCR
Start of differentiation and transfection
(si-IL32, si-ARPP21, si-DNMT1)
Micro-array (IL32)
qPCR (ARPP21 & DNMT1)
ATP assay (IL32 & DNMT1)
Insulin stimulation
C
IL32 qPCR expression
before transfection
si-SCR
si-IL32
D
ARPP21 expression
ARPP21 DNA methylation
DNA Methylation (%)
Myoblasts
Myotubes
cg15778089
cg09866743
cg15459537
E
ARPP21 qPCR expression
F
ARPP21 qPCR expression
si-ARPP21
G
MYOG qPCR expression
TNNI1 qPCR expression
H
SMAD6 expression
SMAD6 DNA methylation
cg12216518
cg17860201
cg27514333
I
PLAC8 expression
PLAC8 DNA methylation
cg01379317
cg08519643
cg27284088
J
75-80% Confluent
85-95% Confluent
Transfection
(si-SMAD6, si-PLAC8)
qPCR and insulin stimulation
K
SMAD6 qPCR expression
si-SMAD6
cMYC qPCR expression
L
PLAC8 qPCR expression
si-PLAC8
TNNI1 qPCR expression

264

Figure S2. Silencing of *ARPP21*, *SMAD6* and *PLAC8* during differentiation of primary human myoblasts

A) mRNA expression of IL-32 in primary human myoblasts (0h) and after 3 and 7 days of differentiation. B) Experimental set-up showing time of transfection with siRNA against *ARPP21*, *IL32* and *DNMT1* with arrows indicating time points of microarray, qPCR, measurements of ATP levels and insulin stimulation in relation to days of differentiation. C) Increased expression of *IL32* during differentiation was significantly blocked with siRNA after 3 and 7 days of differentiation of primary human myoblasts. D) Array data for mRNA expression and DNA methylation (only significant sites) of *ARPP21* in human myoblasts and myotubes (n=13, *q<0.05). E) mRNA expression of *ARPP21* in primary human myoblasts (0h) and after 3 and 7 days of differentiation. F) Increased expression of *ARPP21* during differentiation was significantly blocked with siRNA after 3 and 7 days of differentiation of primary human myoblasts. G) Silencing of *ARPP21* during differentiation resulted in reduced expression of *MYOG* and *TNNI1*. H-I) Array data for mRNA expression and DNA methylation (only significant sites) of *SMAD6* (H) and *PLAC8* (I) in human myoblasts and myotubes (n=13, *q<0.05). J) Experimental set-up for silencing of *SMAD6* and *PLAC8* during myogenesis with arrows indicating time-points of transfection, start of differentiation, qPCR and insulin stimulation. K**)** Silencing of *SMAD6* was confirmed after 1 day of differentiation (2 days after transfection with siRNA) and resulted in significantly reduced expression of *cMYC*. L) Silencing of *PLAC8* was confirmed after 1 day of differentiation (2 days after transfection with siRNA) and resulted in significantly reduced expression of *TNNI1* later during the differentiation (7d). Data are presented as mean ± SEM and analyzed with paired t-test of logged values for qPCR data,

n=4 if nothing else stated, the average of si-SCR at 3d is set to 1 in figure C, F-G and K-L, * $p<0.05$, ** $p<0.01$, ***$p<0.001$ for figure A, D-F and J-K.

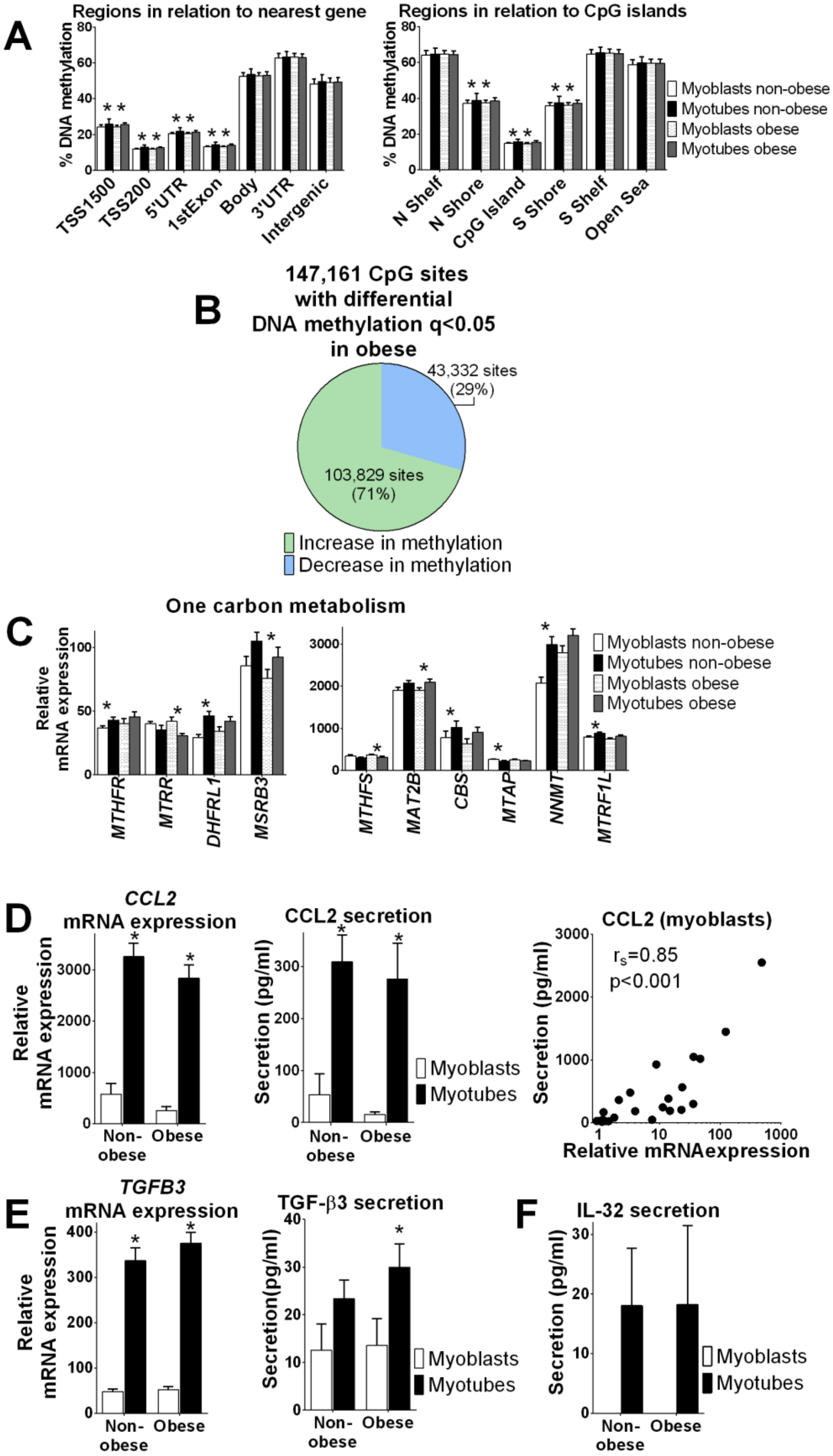
A
Regions in relation to nearest gene
Regions in relation to CpG islands
% DNA methylation
TSS1500
TSS200
5'UTR
1stExon
Body
3'UTR
Intergenic
N Shelf
N Shore
CpG Island
S Shore
S Shelf
Open Sea
Myoblasts non-obese
Myotubes non-obese
Myoblasts obese
Myotubes obese
B
147,161 CpG sites
with differential
DNA methylation q<0.05
in obese
43,332 sites
(29%)
103,829 sites
(71%)
Increase in methylation
Decrease in methylation
C
One carbon metabolism
Relative
mRNA expression
MTHFR
MTRR
DHFRL1
MSRB3
MTHFS
MAT2B
CBS
MTAP
NNMT
MTRF1L
Myoblasts non-obese
Myotubes non-obese
Myoblasts obese
Myotubes obese
D
CCL2
mRNA expression
Relative
mRNA expression
Non-
obese
Obese
CCL2 secretion
Secretion (pg/ml)
Myoblasts
Myotubes
CCL2 (myoblasts)
r$_s$=0.85
p<0.001
Secretion (pg/ml)
Relative mRNAexpression
E
TGFB3
mRNA expression
Relative
mRNA expression
Non-
obese
Obese
TGF-β3 secretion
Secretion(pg/ml)
Myoblasts
Myotubes
F
IL-32 secretion
Secretion (pg/ml)
Myoblasts
Myotubes
Non-
obese
Obese

Figure S3. Differences in methylation, transcription and secreted cytokine levels before versus after differentiation of primary human myoblast from obese and non-obese subjects

A) The average degree of DNA methylation of all analyzed CpG sites in myoblasts versus myotubes for non-obese and obese subjects in different gene regions and in relation to CpG islands. (n=14, *q<0.05). B) A pie chart showing the number and proportions of individual CpG sites with increased and decreased methylation respectively in human myoblasts compared with myotubes (q<0.05) from obese subjects. C) mRNA expression of enzymes in the one carbon metabolic pathway with differential expression in *either* non-obese or obese subjects during myogenesis (n=13, *q<0.05). D) *CCL2* mRNA expression in and secretion from myoblasts and myotubes of non-obese (n=13) and obese (n=14) subjects followed by a correlation between CCL2 secretion and mRNA expression in myoblasts.

E) *TGFB3* mRNA expression in and secretion from human myoblasts and myotubes of non-obese (n=6) and obese (n=6) subjects.

F) IL-32 secretion from human myoblasts and myotubes of non-obese (n=12) and obese (n=13) subjects.

Data are presented as mean ± SEM, * p<0.05 for figure D-F, r_s Spearman correlation coefficient.
